# Biofunctionalized polymer semiconductors toward soft and stretchable transistor-based biosensors

**DOI:** 10.1126/sciadv.aec2641

**Published:** 2026-06-05

**Authors:** Chuanzhen Zhao, Qianhe Liu, Jia-Yuan Chang, Aditri Patil, Lukas Michalek, Yilei Wu, Yujia Yuan, Rachael K. Mow, Yuran Shi, Yating Yao, Kuang-Jung Hsu, Yu Zheng, Zhenan Bao

**Affiliations:** ^1^ Department of Chemical Engineering, Stanford University, Stanford, CA 94305, USA.; ^2^ Department of Electrical Engineering, Chang Gung University, Taoyuan City, Taiwan.; ^3^ Department of Electrical Engineering, Stanford University, Stanford, CA 94305, USA.; ^4^ Department of Chemistry, Stanford University, Stanford, CA 94305, USA.

## Abstract

Organic materials with tunable chemical and mechanical properties are ideal for interfacing with skin and tissue in biomedical applications. While polymer semiconductors (PSCs) have advanced toward skin-like mechanical performance, the limited capacity for biofunctionalization has restricted their biosensing applications. In this study, we introduce a direct biofunctionalization strategy for PSCs based on thiol-ene chemistry. We selectively grafted thiolated biomolecules (e.g., aptamers) onto elastomeric domains within an interconnected semiconductor/elastomer network. This approach enables high-resolution patterning down to 10 micrometers while preserving the electronic performance of PSCs. Leveraging this platform, we designed and fabricated skin-like electrolyte-gated organic field-effect transistors with biofunctionalized channels. These soft and stretchable devices exhibit stable operation in physiological buffers for more than 50 days and maintain performance under up to 50% strain. When functionalized with cortisol-binding aptamers, the sensors achieved sensitive detection across physiologically relevant concentrations, down to the picomolar range. This work establishes a foundation for integrating stretchable and biofunctional PSCs into skin-like wearable devices.

## Teaser

Selective functionalization in polymer semiconductor blends enables bioreceptor grafting for chemical biosensing.

## INTRODUCTION

Bioelectronic devices that can interact and interface with tissues and organs to record clinically relevant signals are important for health monitoring applications (*1*–*8*). Recently, soft and stretchable bioelectronics based on polymeric materials have emerged as promising platforms for biomedical applications due to their compatibility with biological systems (*9*–*11*). A core building block of these devices is the organic field-effect transistor (OFET), which can maintain high electrical performance under skin- or tissue-like deformations (typically tens of percent) (*12*–*14*). For example, skin-inspired OFETs and circuits have been developed to record, amplify, and digitize biophysical signals such as pressure and temperature measured directly on skin (*13*, *15*–*17*). Extending OFETs toward biochemical sensing, however, requires functional interfaces that can directly interact with analytes in physiological environments. Thus, to advance the next generation of human-integrated biomedical devices, it is essential to develop soft and stretchable OFETs with stable and chemically tunable interfaces.

Different approaches for direct attachment of biorecognition molecules on the polymer semiconductor (PSC) channel have been explored for achieving active sensing layers for various biomedical applications, including chemical biosensing (*18*–*21*), tissue adhesion (*10*), and immune compatibility (*18*, *22*). This architecture can enable efficient amplification and transduction of small potential and charge changes around the channel (*23*–*25*). However, there exist limited semiconductor biofunctionalization strategies with reliable performance. Physisorption strategies, such as electrostatic force, hydrogen bonding, and π-π interactions, suffer from long-term instabilities and cause OFET drifting due to detachment of these functional groups (*26*, *27*). On the other hand, covalent chemical modifications are challenging due to the inherent lack of reactive functional groups on most organic semiconductors (OSCs) (*19*, *23*). Existing strategies for direct modification use backbone or side-chain engineering approaches, which are synthetically demanding and limit scalability and accessibility in biosensing applications (table S1) (*19*, *20*, *28*). Other approaches require harsh and nonspecific surface treatments, such as oxygen plasma, to generate reactive groups, which introduce charge traps that damage their electronic performance (*29*, *30*). Therefore, reliable and minimally disruptive covalent biofunctionalization strategies are crucial for overcoming these limitations and unlocking broader biosensing applications.

**Fig. 1.**
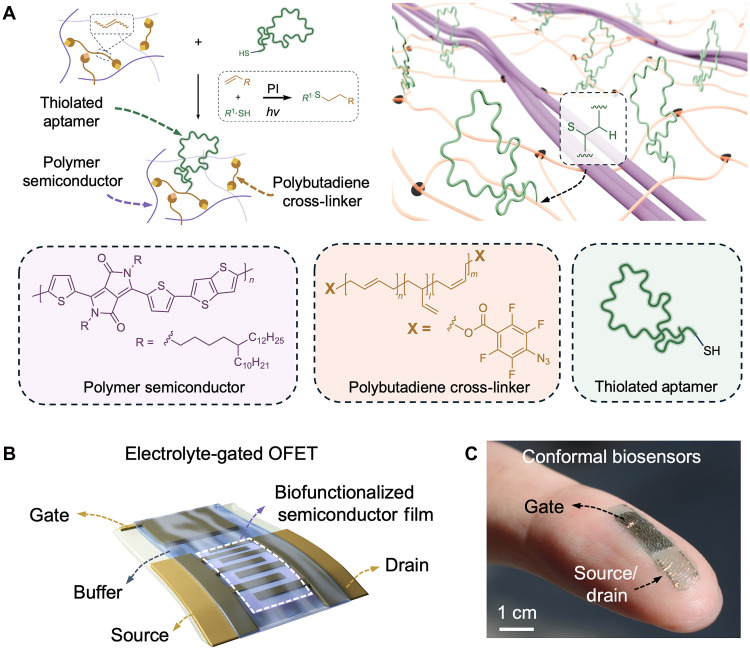
Schematic of biofunctionalization strategies on PSCs. (**A**) Schematic illustration of covalent functionalization of poly-thieno[3,2-*b*]thiophene-diketopyrrolopyrrole (DPPTT)/perfluorophenyl azide end-capped polybutadiene (BA) interconnected polymer network with thiolated aptamers through thiol-ene reaction. (**B**) Schematic of soft and stretchable EG-OFETs. (**C**) Photo of a skin-like conformal EG-OFET biosensor on a fingertip. PI, photoinitiator.

In this study, we present a strategy for covalently functionalizing biorecognition elements (e.g., aptamers) on PSCs to achieve biofunctionalities and mechanical softness simultaneously. We used an interconnected PSC/elastomer network, in which the electronically active and chemically reactive components are spatially distinct (Fig. 1A). Using selective thiol-ene click chemistry, thiolated aptamers are covalently grafted to the elastomer backbone, which avoids direct modifications to the electronically active semiconductor components, while their proximity enables close biomolecule-OSC interactions for sensitive ionic-electronic coupling. This approach enables high-resolution (<10 μm) photopatterning of aptamers and aptamer-functionalized semiconductors with stable electronic performance in buffer for more than 50 days. We further designed and fabricated soft, stretchable electrolyte-gated (EG)–OFET biosensors that operate reliably up to 50% mechanical strain (Fig. 1, B and C). Together, our material functionalization strategy and device design enable soft biosensors with cortisol detection sensitivity down to picomolar concentration.

## RESULTS

### Covalent functionalization of aptamers on OSCs

Direct modification of biorecognition molecules in soft OFET-based biosensors can be achieved either through functionalization on stretchable semiconductors or via gate layer modification using extended-gate capacitively coupled architectures. Among these approaches, direct functionalization of the active sensing layer provides an effective strategy for achieving optimal sensitivity without relying on complex device designs for gate coupling or additional fabrication steps. Previous studies have demonstrated that PSC/elastomer networks can achieve intrinsic stretchability while retaining electrical performance comparable to that of the neat PSC (*31*). In such systems, brittle PSCs are blended and/or cross-linked with thermoplastic elastomers [e.g., styrene-ethylene-butylene-styrene (SEBS) and polybutadiene] to form interconnected fibrous morphologies that enhance stretchability (*31*, *32*). If nanoconfinement of PSC is achieved, there could even be a boost in charge carrier mobility despite blending with a large fraction of insulating elastomers (*31*). We hypothesized that biofunctionalization of the inactive elastomer domains in this nanoscale phase-separated network would promote close interactions between the biorecognition element and the semiconductor. As a result, biomolecule-binding–induced potential changes can modulate charge transport in the semiconductor channel and, therefore, alter OFET electrical response.

To test this hypothesis, we used a two-component semiconductor/elastomer thin film in which only the elastomer phase contains reactive C═C sites. Here, poly-thieno[3,2-*b*]thiophene-diketopyrrolopyrrole (DPPTT) was selected as the PSC due to its state-of-the-art charge transport properties, while the elastomer matrix was formed using perfluorophenyl azide end-capped polybutadiene (BA) cross-linkers with reactive polybutadiene backbones (*32*). OFETs with DPPTT/BA channels have demonstrated high stretchability (100%), high charge carrier mobility (up to 1 cm^2^ V^−1^ s^−1^), and high cycling stability up to 5000 stretch-release cycles (*32*). Choosing BA as the elastomeric phase would simultaneously enable stretchability and selective grafting of thiolated biomolecules (e.g., thiolated aptamers) onto its C═C bonds via ultraviolet (UV)–activated thiol-ene click chemistry. Because of the nanoscale phase separation formed by the DPPTT/BA cross-linked network (*32*), we anticipate effective modulation of DPPTT charge transport in response to conformational changes of the aptamer. Throughout this functionalization process, the semiconductor component (DPPTT) remains chemically inert, minimizing disruption to its charge transport properties.

**Fig. 2.**
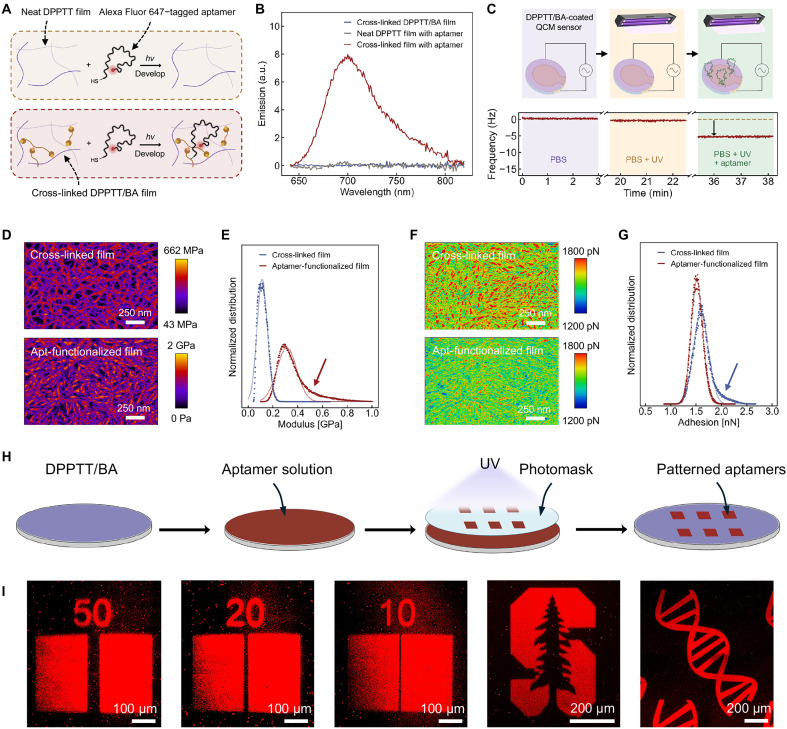
Characterizations of aptamer functionalization and patterning on DPPTT/BA surface. (**A**) Schematic of functionalization of Alexa Fluor 647–tagged aptamers on neat DPPTT and DPPTT/BA thin films. (**B**) Fluorescence spectra of Alexa Fluor 647–tagged aptamers functionalized onto neat DPPTT (gray) and DPPTT/BA (red) thin films with control spectra of DPPTT/BA thin film without aptamer functionalization (blue). λ_ex_ = 600 nm. a.u., arbitrary units. (**C**) Schematic (top) and frequency change (bottom) during real-time functionalization of thiolated aptamers onto DPPTT/BA monitored using quartz crystal microbalance (QCM). (**D** to **G**) Nanomechanical mapping of Derjaguin-Muller-Toporov (DMT) modulus [(D) and (E)] and adhesion force [(F) and (G)] of DPPTT/BA thin films and aptamer-functionalized DPPTT/BA films, respectively. The arrow in (E) indicates the high-modulus region after aptamer functionalization, and the arrow in (G) indicates the high-adhesion BA-domain region in the network. Apt, aptamer. (**H**) Schematic illustrations of the photopatterning process of thiolated aptamers on DPPTT/BA thin films. Created in BioRender. C.Z. (2026); https://BioRender.com/ykesoj2. (**I**) Fluorescence microscope images of patterned aptamers with 50-, 20-, and 10-μm feature sizes, and complex patterns such as the Stanford logo and the double-stranded DNA structure.

We first confirmed the reactivity of thiolated aptamers on the cross-linked semiconductor thin film by monitoring fluorophore-labeled aptamers (thiol modified on the 5′ end, Alexa Fluor 647 modified on the 3′ end) with fluorescence spectroscopy (Fig. 2A; Methods). Following aptamer functionalization on cross-linked DPPTT/BA films, an emission peak at ∼700 nm was observed, whereas no peak was detected on neat DPPTT films that were processed identically (Fig. 2, A and B). This result confirms that effective aptamer grafting occurs only in the presence of reactive C═C bonds in the elastomer component, validating our selective grafting strategy.

To validate the functionalization process as a UV-triggered reaction, we performed in situ grafting studies using a quartz crystal microbalance (QCM) with a flow cell (fig. S1 and Methods). DPPTT/BA-coated sensors exposed either to UV light (∼365 nm) under phosphate-buffered saline (PBS) flow or thiolated-aptamers-in-PBS flow without UV showed only minimal frequency shifts after returning to PBS flow (fig. S2, A to C), indicating minimal mass (i.e., aptamer) uptake through physical adsorption. Sensors exposed to simultaneous UV light and aptamers-in-PBS flow exhibited a frequency decrease of ∼6 Hz upon returning to PBS flow, corresponding to an aptamer surface density of ∼0.047 aptamers/nm^2^ (Fig. 2C and note S1)—comparable to values (∼0.02 to 0.06 aptamers/nm^2^, measured via chronocoulometry) reported in electrochemical aptamer-based biosensors with aptamers grafted on an Au surface (*33*). To rule out the possibility of aptamer physisorption, we showed that DPPTT/BA-coated sensors exposed to acrydite-functionalized aptamers in PBS showed minimal mass changes after UV exposure. These results show that thiolated aptamer grafting can only occur under UV exposure and with reactive thiol groups, confirming the light-triggered nature of our covalent grafting methods. Furthermore, contact angle measurements showed an increase in hydrophilicity after aptamer functionalization, with an average contact angle of 101.1° ± 0.4° dropping to 90.1° ± 2.8°, resulting from the hydrophilic aptamers on the surface (fig. S3).

To study the film’s mechanical properties after aptamer functionalization, we performed nanomechanical mapping using atomic force microscopy (AFM) (Fig. 2, D to G, and fig. S4). Cross-linked DPPTT/BA films exhibited a fibrous morphology due to nanoscale phase separation between DPPTT and BA (Fig. 2D, top) (*32*). After aptamer functionalization, the film morphology was retained (Fig. 2D, bottom). An increase in average film modulus was observed, likely due to partial collapse of the film from solvent exposure [i.e., isopropyl alcohol (IPA)] during processing (Fig. 2E). In addition, a high-modulus shoulder (arrow in Fig. 2E) appeared in the distribution, indicative of local stiffening of the elastomer matrix. A similar effect was seen in the adhesion distribution. The cross-linked film showed a right-skewed distribution with a shoulder at elevated adhesion [high-adhesive areas (red) in Fig. 2F and arrow in Fig. 2G]. After aptamer functionalization, however, the adhesion shoulder disappeared, which we attribute to weaker AFM tip–aptamer interactions compared to the stronger tip-elastomer interactions before functionalization (*34*).

An exciting opportunity that the UV-triggerable biografting process offers is photopatterning capability. High-resolution patterning of biomolecules is crucial for various biomedical applications, including multiplexed sensing, miniaturized biological assays, and drug screening (*35*–*37*). Existing biomolecule patterning techniques, such as soft lithography–based microcontact printing, are often limited to specific materials (e.g., polydimethylsiloxane and Au) due to substrate-specific interactions, thereby restricting their compatibility with organic surfaces and potential for scalable and complex patterning (*38*, *39*). Our covalent functionalization approach enables precise photopatterning of biomolecules on cross-linked DPPTT/BA surface using standard photomasks, which is compatible with standard aligners commonly used in conventional photolithography (Fig. 2H). Upon UV exposure, thiolated aptamers in the exposed regions covalently react with available C═C sites on the cross-linked film, while those in the unexposed regions are removed during solvent development (Fig. 2H). Aptamers can be patterned into different features on a square centimeter scale (∼1.5 cm by 1.5 cm) with a resolution down to 10 μm (Fig. 2I and fig. S5).

### Aptamer-functionalized OFETs with record-high stable performance in buffer

**Fig. 3.**
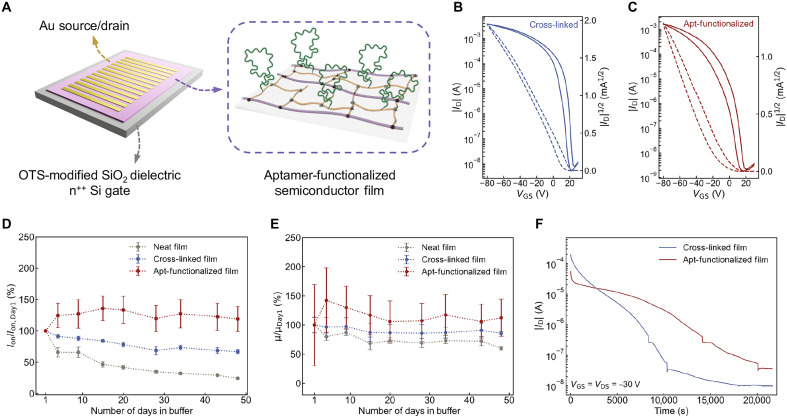
Characterization of electrical performance of aptamer-functionalized OFETs. (**A**) Schematic of device structure of bottom-gate, top-contact (BGTC) rigid OFETs with DPPTT/BA channel functionalized with aptamers. The schematic illustration on the right shows the polymer structures of aptamer-functionalized DPPTT/BA. (**B** and **C**) Representative transfer curves of cross-linked DPPTT/BA (B) and aptamer-functionalized (C) BGTC transistors. Solid lines represent the *I*_D_, and dotted lines represent the square root of the *I*_D_. Transfer curves were collected at *V*_DS_ = −80 V. (**D** and **E**) Time evolution of on-currents (D) and mobilities (E) of OFETs immersed in buffer under ambient conditions. Comparisons were made with neat DPPTT, cross-linked DPPTT/BA, and aptamer-functionalized DPPTT/BA OFETs immersed in PBS for 48 days. Data in (D) and (E) are presented as mean values, and the error bars represent the SD from *N* = 4 devices. (**F**) Bias stress stability of OFETs with cross-linked DPPTT/BA and aptamer-functionalized DPPTT/BA. Transistors were under constant application of *V*_GS_ = *V*_DS_ = −30 V in air for 1 hour.

We next tested the electrical performance of aptamer-functionalized DPPTT/BA films in OFETs (Fig. 3A). These OFETs were fabricated in a top-contact/bottom-gate structure with octadecyltrimethoxysilane (OTS)–modified SiO_2_ dielectric with n^++^ Si gates. Representative transfer curves of solid-state DPPTT/BA transistors with or without aptamer functionalization yielded similar electrical performance, characterized by similar mobilities, threshold voltages, and on/off ratios (∼10^6^) (Fig. 3, B and C). The aptamer-functionalized DPPTT/BA thin film maintained good electrical properties with an average mobility of 0.66 ± 0.20 cm V^−1^ s^−1^ (55% of the values of DPPTT/BA thin films). Aptamers, as charged molecules, are expected to have an impact on the charge transport of OSCs, as reported in other organic and inorganic semiconductor systems (*40*). These variations were shown to have less than an order of magnitude impact on mobility in aptamer-functionalized OFETs. A slightly larger hysteresis and threshold voltage shifts were observed after aptamer functionalization, which is also attributed to the introduction of water molecules in the PSC surface as traps during the functionalization process (Fig. 3, B and C).

To study the long-term stability of OFETs in buffer, we incubated OFETs in a physiological buffer (artificial sweat) for 48 days and monitored their electrical properties during incubation. Our aptamer-functionalized devices maintained their on-current and retained ∼113% (two-tailed *P* ∼0.76, indicating no statistically significant change) of their original mobility after 48 days of incubation in the buffer (Fig. 3, D and E, and fig. S6), which, to the best of our knowledge, is among the longest stable performances of nonencapsulated OFETs in a buffer environment (>1000 hours) (*41*, *42*). In contrast, the OFETs with neat DPPTT channels showed >80% decrease in on-current after 48-day incubation (Fig. 3D). We hypothesized that the high cross-link density of the DPPTT/BA network and the additional protection from aptamers’ charged backbones contributed to the high stability. In addition to long-term stability, we also studied the effect of aptamer functionalization on the semiconductor’s short-term bias-stress stability. With continuous bias over 30 min, the aptamer-functionalized devices exhibited similar stability as the cross-linked films (Fig. 3F). While both DPPTT/BA and aptamer-functionalized DPPTT/BA films showed bias-stress drifting, which is a common phenomenon in OFETs when biased at high voltages (i.e., −30 V), the impact of functionalization on device performance is negligible during prolonged incubation in buffer. These findings demonstrate that aptamer-functionalized OFETs exhibit superior environmental and operational stability in buffer, with the functionalization process having minimal impact on the semiconductor’s electrical performance and device operation.

### Soft and stretchable EG-OFET biosensors

**Fig. 4.**
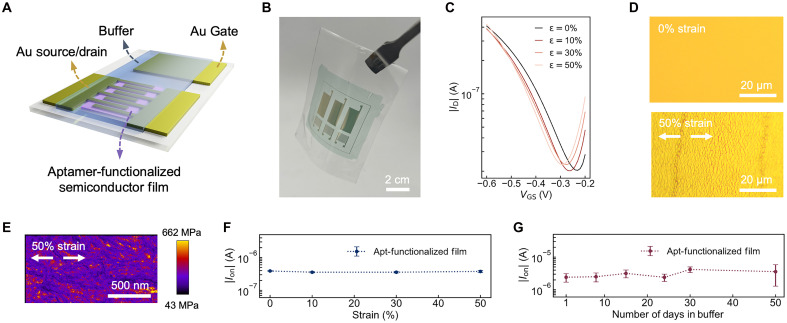
Soft and stretchable electrolyte-gate EG-OFETs. (**A**) Schematic of EG-OFET device with aptamer-functionalized semiconductor film. The aptamer-functionalized DPPTT/BA semiconductor channel, source/drain electrodes, and side gate are in direct contact with the buffer. (**B**) A photo of a fabricated soft and stretchable EG-OFET. (**C**) Transfer characteristics of aptamer-functionalized EG-OFET under 0, 25, and 50% mechanical strain along the channel direction. Transfer curves were collected at *V*_DS_ = −0.4 V. (**D**) Microscope images of Au electrodes of EG-OFETs under 0% (top) and 50% (bottom) strain. (**E**) Atomic force microscope images of modulus mapping of aptamer-functionalized DPPTT/BA films under 50% strain (E). (**F**) The on-current changes of soft EG-OFET when stretched up to 50%. Data are presented as mean values, and the error bars represent the SD from five devices. (**G**) Time evolution of on-currents of aptamer-functionalized EG-OFETs immersed in buffer. EG-OFETs were immersed in buffer for 50 days. Data are presented as mean values, and the error bars represent the SD from three devices.

Biofunctionalized semiconductor can serve as the active channel in EG-OFETs for sensitive and selective biosensing (*23*, *26*, *43*, *44*). In EG-OFET biosensors, the OSC channel is in contact with electrolyte, allowing for close interactions between bioreceptors on OSCs and analytes in the electrolyte for signal transduction and amplification (Fig. 4A). Upon gate biasing, mobile ions in the electrolyte accumulate at both OSC and gate surfaces to form electrical double layers, which act as high-*k* (e.g., *k* = ∼80 for water) and ultrathin (e.g., Debye length < 1 nm in 1× PBS) dielectrics (fig. S7) (*23*, *45*). As a result, EG-OFETs can be operated at low voltages (<1 V), making them ideal for wearable or implantable applications (note S2). Using our aptamer-functionalized DPPTT/BA polymers, we developed soft and stretchable EG-OFETs as an ideal biosensing platform on the skin and tissues.

To achieve desirable mechanical properties while maintaining high electrical performance, we selected an elastomer [i.e., styrene-butadiene-styrene (SBS)] as substrate and microcracked Au as electrodes for stretchable EG-OFETs (Fig. 4, A and B). Microcracked Au electrodes have been reported using metal evaporation on elastomers, and such electrodes were shown to maintain high conductivity (sheet resistance of <35 Ω square^−1^) when stretched >100% (*46*, *47*). This design enabled EG-OFETs with low voltages (<400 mV) and stretchability up to 50% with minimal changes in their electrical performance (Fig. 4C). We also validated that these EG-OFETs were operational in biologically relevant buffer systems—artificial sweat and artificial saliva, highlighting their promise for future in vivo applications (fig. S8). Under 50% strain, we observed microcracked Au structures (Fig. 4, D and E) and minimal changes in aptamer-functionalized semiconductor morphology (Fig. 4E). The channel current remained stable under strains up to 50% (Fig. 4F and figs. S9 and S10). Furthermore, EG-OFETs often suffer from electrical degradation when incubated in buffer without encapsulation, with stable performance lasting only from a few hours to a month in buffer (*48*). Our aptamer-functionalized EG-OFETs maintained stable long-term and short-term electrical performance in buffer for a record high of 50 days and over 1 hour of continuous bias stress without major degradation, attributed to the high stability of the cross-linked DPPTT/BA films and the grafted aptamers (Fig. 4G, and figs. S11, B and C, to S14).

Aptamer-based FET biosensors have previously shown high sensitivity and selectivity for detecting small molecules, such as serotonin, dopamine, cortisol, and phenylalanine in physiological environments (*45*, *49*–*51*). In these In_2_O_3_-based sensors, biomarker binding induces a conformational change in the aptamers, which alters local ion distributions and, consequently, modulates the surface potential of the semiconductor. Such devices have been used in applications ranging from implantable neural probes for neurotransmitter monitoring to wearable devices for biomarker detection in sweat (*49*, *50*).

**Fig. 5.**
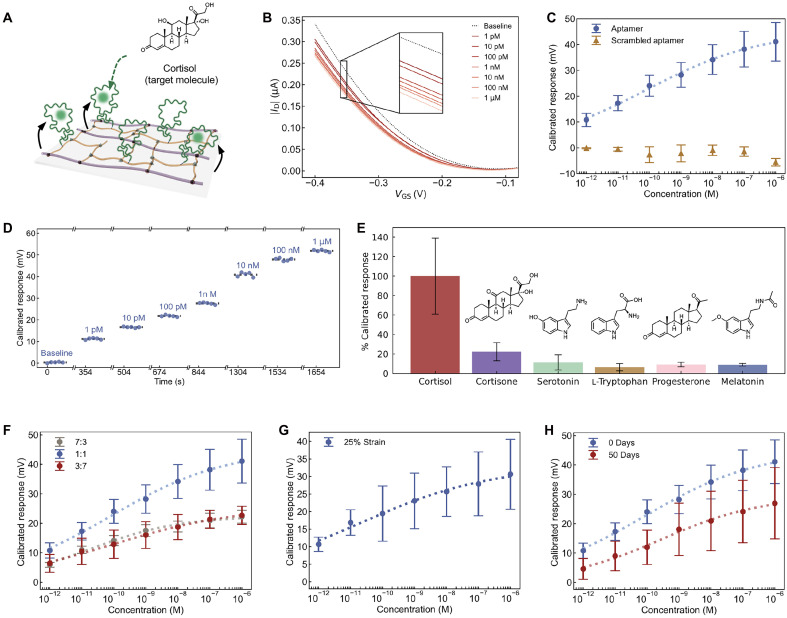
Cortisol sensing using aptamer-functionalized electrolyte-gate EG-OFETs. (**A**) Schematic of the sensing mechanism of cortisol-specific aptamer-functionalized EG-OFETs based on conformational changes of aptamers. (**B**) Transfer characteristics of aptamer-functionalized EG-OFETs with different cortisol concentrations from 1 pM to 1 μM in buffer (PBS). (**C**) Calibrated responses to cortisol for EG-OFETs functionalized with cortisol aptamer (blue) or scrambled sequence (yellow). All error bars represent SDs (*N* = 5 devices with cortisol aptamer, *N* = 3 devices with scrambled sequence). (**D**) Real-time response of aptamer-functionalized EG-OFETs to increasing aptamer concentration in buffer. (**E**) Aptamer-functionalized EG-OFET responses to cortisol versus nontargets in buffer. All error bars represent standard means (*N* = 5 devices with cortisol responses, *N* = 3 devices with interference targets). (**F**) Calibrated responses to cortisol for aptamer-functionalized EG-OFETs with different ratios of DPPTT:BA. (**G**) Calibrated responses to cortisol for aptamer-functionalized EG-OFETs under 25% strain. (**H**) Calibrated responses to cortisol for aptamer-functionalized EG-OFETs before and after 50 days of immersion in PBS. All error bars represent standard means with *N* = 3 devices [(F) to (H)].

In our EG-OFET biosensor, as a proof of concept, we selected a well-characterized cortisol-binding aptamer (*50*) as the biorecognition moiety, grafted it onto the DPPTT/BA semiconductor layer, and tested its cortisol-sensing performance (Fig. 5A). DPPTT has hydrophobic backbones and side chains, which limits water permeability into the channel. As a result, the sensing mechanism is dominated by surface-potential modulation or channel depletion, analogous to that observed in inorganic semiconductors such as In_2_O_3_ (*45*, *49*). In the aptamer-functionalized EG-OFET, cortisol-induced conformational changes occur in negatively charged aptamer phosphodiester backbones in conjunction with the rearrangement of associated solution ions. As previously report, cortisol aptamers are hypothesized to move away from the surface upon target binding, thereby depleting surface positive charges and reducing current in p-type semiconductors (*45*, *49*). Our aptamer-functionalized EG-OFETs successfully detected cortisol concentrations over a broad dynamic range, from 1 pM to 1 μM, encompassing the full physiologically relevant range in human perspiration (Fig. 5, B and C). The real-time response plots showed that the EG-OFETs are stable within each concentration measurement (plotted five consecutive scans) and gave distinct responses between concentrations (Fig. 5D). To model the aptamer-cortisol binding behavior, we fit the calibrated responses of the cortisol sensor using the Hill equation to extract the sensor’s limit of detection (LOD), maximum sensitivity, maximum response, cortisol-binding affinity, apparent cooperativity, and device-to-device variations (table S2, eq. S1, and fig. S15). Our aptamer-functionalized EG-OFET device exhibited a maximum response of 50.57 mV with a Hill coefficient of 0.26, indicating detection of cortisol within the physiologically relevant range. It demonstrated a maximum sensitivity of 7.54 mV per decade across a dynamic range spanning 0.38 pM to 17.1 nM, with a LOD < 1 pM. As a control, our sensors functionalized with a scrambled cortisol aptamer sequence—containing the same number of bases as the cortisol aptamer sequence—produced negligible response across the same cortisol concentration range (Fig. 5C). The sensors exhibited high selectivity against structurally similar and potentially interfering molecules present in physiological environments (Fig. 5E and fig. S16). Cortisol (10 nM) and interface molecules [10 nM (cortisone) or 1 nM (others)] were measured in their physiological concentrations in sweat. The dynamic range is similar to that reported in inorganic transistor biosensors, and the amplitude of response is ∼2-fold of that reported in inorganic systems (*50*) (table S3).

Leveraging this effective cortisol-sensing platform, we investigated how the ratios of charge transport material (DPPTT) and biorecognition elements (aptamer grafted onto BA) are reflected in the sensor behavior. We hypothesized that a nanoscale phase-separated DPPTT/BA network could allow aptamers tethered on BA to modulate the charge transport of DPPTT, whereas excessive BA content will have tethered aptamers further from the DPPTT domains, thereby diminishing their influence on channel modulation. To test this hypothesis, we evaluated the sensitivity and response of cortisol sensors fabricated with varying ratios of DPPTT/BA (DPPTT:BA; 7:3, 1:1, and 3:7 mass ratios) (Fig. 5F and table S2). When the BA fraction was reduced from 50% (used in our benchmark cortisol sensor) to 30%, the device’s sensitivity and maximum response decreased by ∼50%, consistent with a lower aptamer grafting density. However, increasing the BA fraction from 50 to 70% also reduced sensitivity and the maximum response to ∼50% relative to our benchmarked sensor despite the higher aptamer density. We attribute this nonmonotonic behavior to the formation of larger BA-rich domains that has a large fraction of aptamers spatially isolated from the charge transport material (fig. S17). As a result, binding events on these aptamers are not close enough to affect charge transport in DPPTT fibers and contribute only weakly to channel modulation despite their increased abundance. Together, these results demonstrate the delicate interplay of nanoscale morphology and electronic coupling between aptamers and DPPTT, rather than aptamer quantity alone, which governs the sensor performance.

We also tested the mechanical and environmental stability of our aptamer-functionalized EG-OFET. At under 25% strain, our sensors showed a response and sensitivity similar to those under 0% strain (Fig. 5G and table S2). Furthermore, after incubation for 50 days in buffer, our sensors also demonstrated cortisol-sensing response and sensitivity with no significant decrease compared to that of the as-fabricated devices (Fig. 5H). We attribute this long-term aptamer stability to the strong covalent linkages between the elastomers and aptamers. In contrast, most existing aptamer-based sensors rely on Au─S bonds, which are comparatively less stable. Last, we validated these EG-OFET sensors in various biologically relevant buffers, such as artificial sweat, to help assess sensor performance in the presence of protein and ionic interferents (fig. S18A). We also performed cortisol sensing with different aptamer grafting densities on the surface. The results showed that maximum response sensitivity (as in millivolts per decade) to cortisol increased from ∼4.4 to ∼9.7 mV per decade with increasing aptamer concentrations (fig. S18B and table S2). The LOD and the dynamic range remain similar across different aptamer densities (table S2 and fig. S18C). Across all conditions, the sensors exhibited LOD below 10 nM, comparable to that obtained from benchmarked transistor-based cortisol sensors (table S3) (*50*, *52*, *53*). Together, these soft aptamer-functionalized EG-OFETs combine high sensitivity and selectivity with excellent mechanical compliance and stability, highlighting their potential for future wearable biosensing applications.

## DISCUSSION

In this work, we present a covalent functionalization strategy that enables the development of intrinsically stretchable, skin-conformal OFET-based bioelectronics. By selectively grafting thiolated aptamers onto the elastomeric domains of semiconductor/elastomer cross-linked films via thiol-ene chemistry, our approach achieves robust and spatially controlled biofunctionalization without compromising the electronic performance of the PSC. Aptamer grafting onto reactive C═C bonds within the BA phase of a model DPPTT/BA system enabled high-resolution (<10 μm) photopatterning and stable semiconductor operation in physiological buffer for over 50 days. This aptamer functionalization approach led to soft and stretchable EG-OFET biosensors capable of detecting cortisol with high sensitivity (down to picomolar levels), excellent selectivity, and reliable performance up to 50% strain. While thiolated aptamers served as the model biorecognition element in this study, this strategy can also be used with other cross-linking chemistries (e.g., azide/C─H, acrylate/acrylate, and 1-ethyl-3-(3-dimethylaminopropyl)carbodiimide/*N*-hydroxysuccinimide), bioreceptors (e.g., enzymes and antibodies), and PSC/elastomer networks. In addition, future studies that focus on tuning receptor density, improving regeneration, cycling, continuous monitoring, and antibiofouling capabilities, enhancing long-term device stability, and expanding molecular recognition functionalities will expand the platform’s utility. Functionalizing the transistor channel with biomolecules provides exciting opportunities in various biomedical applications, including chemical biosensing, tissue adhesion, and immune compatibility. Overall, our work offers a versatile approach for the direct covalent biofunctionalization of semiconductor thin films, thereby advancing the development of next-generation, skin-inspired biosensors for wearable and implantable applications.

## METHODS

### Materials

DPPTT, BA, and bis(6-((4-azido-2,3,5,6-tetrafluorobenzoyl)oxy)hexyl) decanedioate were synthesized according to reported methods (*32*, *54*). PBS was purchased from Thermo Fisher Scientific Inc. Oligonucleotides (aptamers) were obtained from Integrated DNA Technologies. Poly(SBS) (D1102) with a polystyrene content of 28% was provided by Kraton Polymers. Photoresist AZ 1512 and poly(methyl methacrylate) (PMMA) 495K A4 were obtained from MicroChemicals, and developer MF319 was obtained from Kayaku Advanced Materials. MG Chemicals 415-1L Ferric chloride copper etchant solution was purchased from MG Chemicals. Artificial saliva (1700-0303) and artificial sweat (I2BL-0011) were purchased from Pickering Laboratories Inc. (Mountain View, CA) and used as received. Unless otherwise noted, all other chemicals were purchased from Sigma-Aldrich Co. and were used without further purification.

### DPPTT/BA polymer film preparation

DPPTT (*M*_n_ = 67.4 kg mol^−1^, *M*_w_ = 269.8 kg mol^−1^, Đ = 4.00, 5 mg/ml; anhydrous chlorobenzene) and BA (polybutadiene backbone with *M*_n_ of ∼1200, BA with *M*_n_ of ∼1500; anhydrous chlorobenzene) solutions were mixed [2:1 DPPTT:BA (v/v)] and stirred under nitrogen at 85°C for 10 min. The blend solution was spin coated onto substrates [e.g., OTS-modified Si/SiO_2_ (300 nm), SBS, and Au QCM sensors] at 1000 rpm for 1 min and annealed at 150°C for 1 hour under nitrogen.

### Aptamer functionalization and patterning

Thiolated cortisol-binding aptamers (5′/ThioMC6-D/CGACCGGTCTGGGGACCCTGTCTGGGTGTGTGGGTAGTAGGTCG) and scrambled aptamers (5′/ThioMC6-D/CCACC GCAGTCCGGTCGCTTGCTCGCTGTGTGGGTAGTAGGTCG) were obtained from Integrated DNA Technologies based on previously reported sequences (*50*). In fluorescence patterning experiments, fluorophore tags (Alexa Fluor 647) were added at the 3′-end (5′/ThioMC6/CGACCGGTCTGGGGACCCTGTCTGGGTGTGTGGGTAGTAGGTCGAlexF647N/-3′). All aptamers were obtained with 100 μM solution with high-performance liquid chromatography purification. The aptamer solutions were diluted to 50 μM and mixed with freshly prepared reducing agent [aptamer:tris(2-carboxyethyl)phosphine = 1:1000 (w/w)] and reacted for 1 hour in the dark to reduce the disulfide bonds and generate free thiol groups. Separately, photoinitiator [2-hydroxy-2-methylpropiophenone] was diluted with IPA [1:20 (v/v) for rigid OFET; 1:200 (v/v) for EG-OFET). The reduced aptamer solution was then added dropwise to the diluted photoinititor until a final photoinitiator-to-aptamer ratio of 1:1 (v/v) was reached. The mixed solution was drop casted onto DPPTT/BA surface. A UV-grade quartz was placed on top to spread the solution, followed by UV (E-Series UV-A Blacklight Lamp from Spectro-UV; 365 nm) curing for 15 min (dose of 3600 mJ cm^−2^). After exposure, the film was washed with IPA to remove the aptamer and photoinitiator residues. Then, the surface-functionalized film was annealed in a glovebox at 90°C for 30 min to remove residual solvent. To photopattern aptamers, a chrome-patterned quartz photomask was used in place of the UV-grade quartz plate, while other steps remain the same.

### General characterizations

Optical microscopy images were obtained with a Leica DM4000 M LED microscope. Fluorescence microscope images were taken using a Zeiss LSM 780 confocal microscope. Fluorescence spectra were acquired using a HORIBA Fluorolog-3 spectrofluorometer equipped with a 450-W xenon lamp and a PPD-850 nm photon detection module. All OFET and EG-OFET measurements were conducted using a Keithley 4200 semiconductor parameter analyzer (Keithley Instruments) in air.

AFM images with peak-force nanomechanical mapping mode were collected on the Bruker Dimension Icon AFM with NanoScope 5 controller. ScanAsyst Air (Bruker) AFM cantilever (with a typical resonant frequency of 70 kHz and a force constant of 0.4 N m^−1^) was used (*55*). The images were recorded with a peak force frequency of 2 kHz, an amplitude of 30 nm, a set point of 500 pN, measured with 256 by 256 pixels, and a scan rate of 0.8 Hz. The calibration included the determination of the force constant via thermal tuning *k* ∼ 0.44 N m^−1^, the deflection sensitivity against a sapphire reference sample, and the tip radius *r* of ∼6 nm via tip qualification (NanoScope Analysis 3.0, Bruker) on a rough Ti reference sample. The modulus data were fitted by the Derjaguin-Muller-Toporov (DMT) model, and a Poisson ratio of 0.3 was assumed. The indentation range was around 2 to 4 nm. The measured nanomechanical imaging data were processed using Gwyddion SPM software 2.63.

QCM with dissipation measurements were performed using a Biolin Scientific QSense Explorer and flow module coupled with a peristaltic pump (fig. S1). DPPTT/BA solutions were spin coated onto Au-coated quartz crystal sensors (Phillip Technologies). The temperature of the flow cell was kept at 25°C, and a flow rate of ∼50 μl min^−1^ was used. The grafting density rate was correlated to the mass gain measured via the frequency change of the first-harmonic oscillator of a quartz crystal using the Sauerbrey equation (note S1). Aptamer solutions (100 μM with photoinitiator, as discussed above) and PBS were alternated between measurements. Three conditions were tested: UV+/aptamer– (UV on with PBS in the flow cell), UV−/aptamer+ (UV off with aptamer solution in the flow cell), and UV+/aptamer+ (UV on with aptamer solution in the flow cell).

### Rigid OFET fabrication

Si/SiO_2_ (300 nm) substrates were first modified with a dense OTS monolayer. The modification process was as follows: Si/SiO_2_ substrates were first treated with O_2_ plasma for 3 min. OTS (2.4 mM in trichloroethylene) was drop casted onto the substrate, incubated for 30 s, and spin coated at 2000 rpm for 30 s. The substrate was then vapor annealed in a desiccator purged with ammonium hydroxide solution (28.0 to 30.0% in H_2_O) overnight. The OTS-treated Si/SiO_2_ substrate (typical water contact angle is ∼106° to 109°) was cleaned rigorously with toluene using foam swabs and dried with N_2_ gas. DPPTT/BA films and aptamer functionalization were then prepared as discussed above. Au (40 nm) contacts were thermally evaporated onto the semiconductor film through a shadow mask with *W*/*L* ratios of 65.

### EG-OFET fabrication

For rigid EG-OFET, Si/SiO_2_ (300 nm) substrates were used as is for substrate. DPPTT/BA films and aptamer functionalization were then prepared as discussed above. AZ 1512 was next spin coated at 2500 rpm for 45 s, and the sample was immediately exposed three times at a dose of 40 mJ cm^−2^ using MicroWriter ML3 and then developed in MF319 for 45 s, rinsed with deionized water, and dried with N_2_. Au electrodes (40 nm) was deposited by e-beam evaporator and lifted off in acetone. The channel length and width were 30 and 160,320 μm, respectively. The gate area was 40.3 mm^2^.

For stretchable EG-OFET, Si/SiO_2_ (300 nm) substrates were first treated with O_2_ plasma for 3 min. Dextran (10 wt % in H_2_O) was spin coated onto the Si/SiO_2_ (300 nm) substrate at 1000 rpm for 1 min and then baked at 150°C for 15 min to form a water-soluble sacrificial layer. Next, SBS (80 mg ml^−1^ in toluene) with 4 wt % pentaerythritol tetra(3-mercaptopropionate) and 4 wt % phenylbis(2,4,6-trimethylbenzoyl)phosphine oxide was spin coated at 800 rpm, cured in the glovebox for 5 min, and baked at 150°C for 10 min as a stretchable substrate. DPPTT/BA films and aptamer functionalization were then prepared as discussed above. PMMA 495K A4 (40 mg ml^−1^ in anisole) was spin coated onto the sample at 800 rpm for 80 s and then at 3000 rpm for 20 s. Next, Cu thin film (150 nm) was deposited by e-beam evaporator. AZ 1512 was then spin coated at 2500 rpm for 45 s, and the sample was immediately exposed at a dose of 100 mJ cm^−2^ using MicroWriter ML3, then developed in MF319 for 45 s, and rinsed with deionized water and dried with N_2_. The sample was immersed in FeCl_3_ solution for 5 s to etch Cu [the original Cu etchant, MG Chemicals 415-1L ferric chloride copper etchant solution, was diluted 1:20 (v/v) in deionized water] and washed with deionized water and dried with N_2_. The sample was flood exposed in a UV curing system (365 nm) at a dose of around 120 mJ cm^−2^, then developed in MF319 for 1 min, rinsed with deionized water, and dried with N_2_. The sample was immersed in acetone to etch PMMA in acetone for 1 s, rinsed with IPA for 10 s, and dried with N_2_. Au electrodes (20 nm) was deposited by e-beam evaporator and lifted off in acetone. The channel length and width were 30 μm and 160 an 320 μm, respectively. For aptamer-functionalized EG-OFET, Au electrodes were first functionalized with a layer of perfluorodecanethiol before aptamer functionalization. Thiolated cortisol-binding aptamers were then functionalized onto DPPTT/BA using the procedure described above.

### Cortisol sensing and calibration

Cortisol sensing measurements were performed by sequentially adding different concentrations of cortisol solution, from low to high, while sweeping the EG-OFETs until five overlapped transfer characteristics were achieved. This method followed previous reports in transistor-based biosensors (*45*, *49*, *50*). Next, calibrated responses of cortisol detection were calculated by normalizing the change in current to the local transconductance, as previously reported, yielding a response proportional to shifts in threshold voltage (*50*). Long-term stability was performed at room temperature for 50 days in buffer (PBS).

## Supplementary Materials

### This PDF file includes:

Supplementary Notes S1 and S2 Tables S1 to S3 Equation S1 Figs. S1 to S18 References
