# Supplementary material for: Biofunctionalized polymer semiconductors toward soft and stretchable transistor-based biosensors
Source: Sci Adv. 2026 Jun 5;12(23):eaec2641. doi: 10.1126/sciadv.aec2641 (PMC13240217; doi:10.1126/sciadv.aec2641)
Supplement: Supplementary file 1 — Supplementary Notes S1 and S2 Tables S1 to S3 Equation S1 Figs. S1 to S18 References [file sciadv.aec2641_sm.pdf]

Science Advances
AAAS

Supplementary Materials for

Biofunctionalized polymer semiconductors toward soft and stretchable transistor-based biosensors

Chuanzhen Zhao *et al.*

Corresponding author: Zhenan Bao, zbao@stanford.edu

Sci. Adv. **12**, eaec2641 (2026)
DOI: 10.1126/sciadv.aec2641

This PDF file includes:

Supplementary Notes S1 and S2
Tables S1 to S3
Equation S1
Figs. S1 to S18
References

Supplementary Note 1: Aptamer grafting density calculation.

A frequency change of ~6 Hz was observed (Fig. 2C) when aptamers were grafted on the DPPTT/BA-coated QCM surface. The equation gives a relationship between mass change and frequency shift in QCM:

$$\Delta m = -C_{QCM} \cdot \frac{\Delta f}{n} \quad (1)$$

where Δm represents mass changes, C_{QCM} is a constant related to QCM crystals (C_{QCM} = 17.7 ng cm^{-2} Hz^{-1}), Δf is the measured frequency changes (~-6 Hz), and *n* is the overtone number (*n* = 1, 3, …). Using equation (1), the aptamer density was determined to be ~0.047 aptamers/nm^2.

Supplementary Note 2: Capacitive coupling and working mechanism of electrolyte-gated organic field-effect transistors

In an electrolyte-gated organic field-effect transistor (EG-OFET), electrical double layers (EDLs) form at the interface of the electrolyte and the semiconductor/metal. The capacitance of the EDLs is directly proportional to the ionic strength of the buffer and the electrode area (*24*). The circuit diagram of an EG-OFET can be simplified into the following fig. S6. The relationship between V_{GATE} and V_{OFET} can be simplified as follows:

$$V_{OFET} = \frac{C_{Gate}}{C_{OFET} + C_{Gate}} V_{Gate} \ (2)$$

Where C_{Gate} and C_{OFET} are the capacitance at the gate and semiconductor interface, VOFET represents the actual voltage dropped on the semiconductor interfaces. Therefore, it is important to have C_{Gate}>>C_{OFET} to (1) maximize the voltage drop at the semiconductor interface and (2) minimize the leakage current through the buffer with $V_{OFET} - V_{Gate} \approx 0$.

Table S1. Comparison of covalent surface functionalization strategy on conjugated polymer films

Conjugated polymers	Functionalization strategy	Synthetic requirement	Stability	Bioreceptors and biomarkers demonstrated	Stretchability	Ref.
DPPTT	EDC/NHS chemistry	Yes, side chain engineering	N/A	Enzyme and glucose sensing	Not stretchable	19
p(NDI-T-ZI/EG) Conjugated polyelectrolyte	EDC/NHS chemistry	Yes, side chain engineering	1.5 hr cycling	Aptamers and dopamine sensing	Not stretchable	28
Cyclic disulfide-containing 3,4-propylenedioxyt hiophene	Thiol-ene click chemistry	Yes, side chain engineering	N/A	Enzyme and glucose sensing	Not stretchable	20
DPPTT	Thiol-ene click chemistry	Not required, blend strategy	1 hr bias<br>50 days storage and sensing at R.T.	Aptamers and cortisol sensing	50% strain of electronic performance<br>25% strain for biosensing	This work

Note: Backbone engineering and side-chain engineering of conjugated polymers offer an excellent approach to functionalized polymers with bioreceptors (*19, 20, 28*). However, these systems typically require additional synthetic efforts and may negatively impact the charge transport of high-performing polymers, and limit their broader application in biosensing.

Instead, our approach uses commercially available and well-established high-mobility polymer semiconductors with a secondary, readily available and easily functionalizable elastomer phase, as discussed in our manuscript (Figure 2A). DPPTT is challenging to functionalize without further synthetic effort on backbone or side-chain engineering while maintaining its high charge carrier mobility. The advantage of our method over direct semiconductor modification is the physical decoupling of electronic transport materials from the biorecognition element, which helps preserve the charge transport of the semiconductor. In our previous work, we have shown that proper selection of the elastic matrix can induce nanoconfinement of the polymer semiconductor to maintain and even exceed charge carrier mobility in blend films compared to neat polymer semiconductors (*31, 32, 56*). Additionally, a central aim of our work is to develop bio-functionalized OSC with high mechanical stretchability. This dual function (i.e., biofunction and stretchability) was achieved through our polymer blend strategy, which achieves both functionalization sites and maintaining good electrical transport. From a chemistry perspective, this work developed a new regime to leverage the polymer-elastomer blend to provide different functionalities. From an application perspective, we provided the first example of photopatternable biofunctionalization strategies that exhibit high mechanical stretchability and long-term buffer stability.

Table S2 Summary of the key sensing matrix of EG-OFETs functionalized with cortisol aptamer

Modification	Condition	Maximum Response (mV)	Hill Coefficient	K_d (pM)	Maximum Sensitivity (mV/dec)	Dynamic Range (20-80%)	LOD
	**Benchmark:**<br>Aptamer Conc.: 500 nM<br>DPPTT:BA 1:1<br>Gate Dielectric: PBS<br>Strain: 0%	50.57	0.26	80.6	7.54	0.38 pM – 17.1 nM	63.7 fM
Aptamer Concentration	5 μM	29.9	0.28	110	9.69	0.80 pM – 15.3 nM	1.53 pM
	50 nM	30.3	0.31	127	4.40	1.44 pM – 11.2 nM	5.37 pM
DPPTT:BA Ratio	7:3	22.5	0.31	18.1	3.96	0.19 pM – 1.69 nM	60.9 fM
	3:7	25.6	0.22	89.6	3.27	0.17 pM – 46.7 nM	93.2 pM
Buffer	Artificial Sweat	25.9	0.23	149	3.49	0.39 pM – 55.7 nM	0.21 pM
Strain	25% Strain	33.07	0.21	17.9	3.98	23.7 fM – 13.5 nM	0.12 pM
Intra-device variation	Benchmark	19.16	0.034	64.5	2.73	0.65 pM – 6.55 nM	11.0 fM
Inter-device variation	Benchmark	17.42	0.13	46.4	1.55	0.19 pM – 12.9 nM	30.8 fM

The limit of detection (LOD) of each EG-OFET device was calculated using methods reported from the literature, with variations from device (N = 3) calibrated response and inverse hill equation fitting of the variation (*57*). The calibrated response from each device type was then mapped to the hill function to calculate the sensor's maximum response, hill coefficient, apparent dissociation constant (K_d), maximum sensitivity, and dynamic range (20-80%).

Intra-device variation indicates variations in different properties for devices fabricated on the same wafer. Inter-device variation indicates variations in different properties for devices fabricated on different wafers.

Table S3 Comparison of representative transistor-based cortisol sensing methods

Transducer	Materials	Biorecognition	Detection limit or lowest conc. investigated	Sensitivity	Stability	Stretchability	Max. response range	Ref.
FET	Inorganic (In_2O_3)	Aptamer	1 pM	N/A, estimated to be 2.5 mV/dec	N/A	Not stretchable	22 mV	50
OECT	Polymer (PEDOT:PSS)	Molecular imprint polymer (MIP)	100 pM	2.68 μA/dec	N/A	Not stretchable	~20 μA	53
FET	MOSFET	Antibody	2.7 nM	0.5%/dec	N/A	Not stretchable	N/A, normalized	52
OFET	Polymer (DPPTT/BA)	Aptamer	1 pM	6.3 mv/dec	50 days in PBS	50% strain	45 mV	This work

Equation S1. Hill equation

$$Y = R_{max} \frac{[X]^n}{K_d^n + [X]^n}$$

where Y = calibrated response, R_{max} = maximum response, [X] = cortisol concentration, n = hill coefficient, and K_d = apparent dissociation constant.

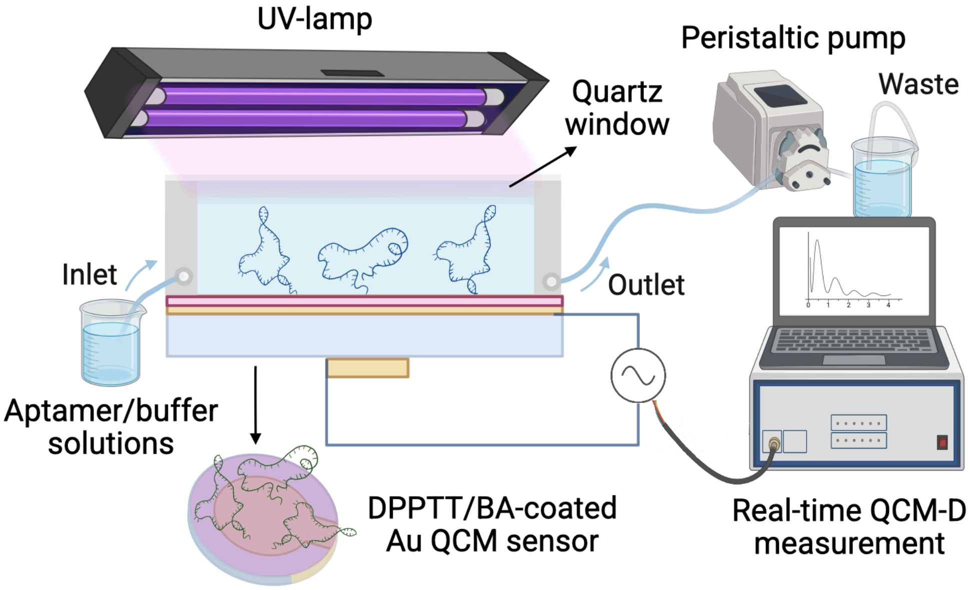

figure S1. Experimental setup of in-situ aptamer grafting density calculation using quartz crystal microbalance with dissipation (QCM-D). The QCM-D is coupled with a peristaltic pump and microfluidic systems. A DPPTT/BA-coated Au QCM sensor was used in the system, featuring a clear quartz window on top for UV-triggered reactions. Real-time QCM-D data were collected while the aptamer and buffer solution were running through the system, as described in the Method. Created in BioRender. Zhao, C. (2026) https://BioRender.com/ykesoj2

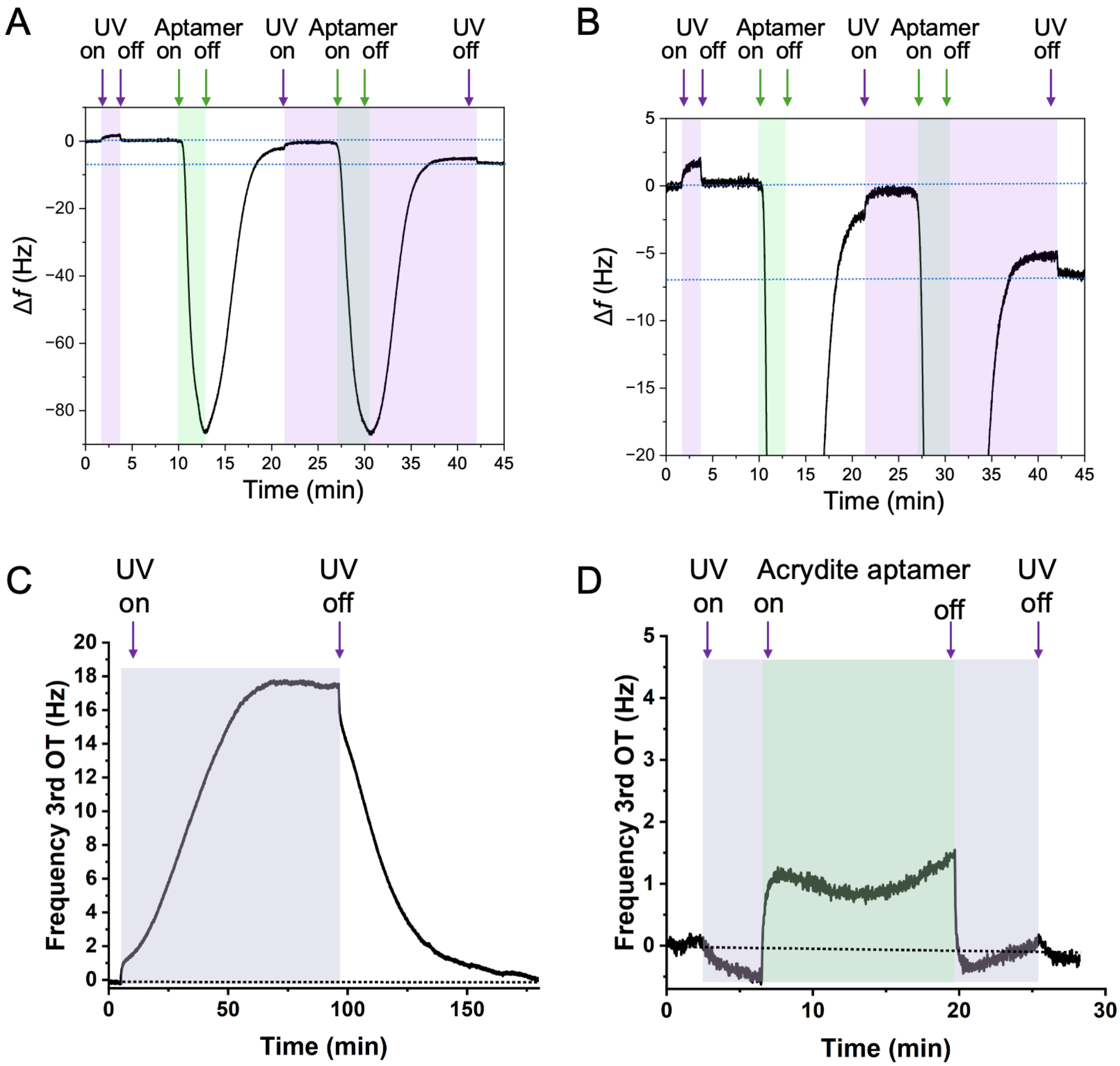

figure S2. Real-time frequency changes of DPPTT/BA-coated QCM sensors with aptamer grafting. (**A**) Frequency changes in QCM-D with UV+/thiolated-aptamer-, UV-/thiolated-aptamer+, and UV+/thiolated-aptamer+ (B) Zoomed-in frequency changes on the DPPTT/BA surface shown in (A). (C) Frequency changes in QCM-D for DPPTT/BA during prolonged (~100 min) UV exposure. (D) Frequency changes in QCM-D with UV+/acrydite-functionalized-aptamer+ (See Method for experimental details).

Note: Prolonged (~100 min) UV exposure was performed on the DPPTT/BA films with PBS flows (fig. S2C). Minimal mass changes were observed before and after the UV exposure, indicating minimal surface property changes induced by the UV light.

It should also be noted that the QCM experimental setup and sensors are different than EG-OFET cortisol sensing experiments, due to the distinct requirement of sample sizes and measurement setup in QCM. Therefore, it is challenging to directly correlate QCM densities with sensing experiments. The QCM experiments provide fundamental insights into in situ grafting and served as a reference for the aptamer grafting density on the DPPTT/BA surface.

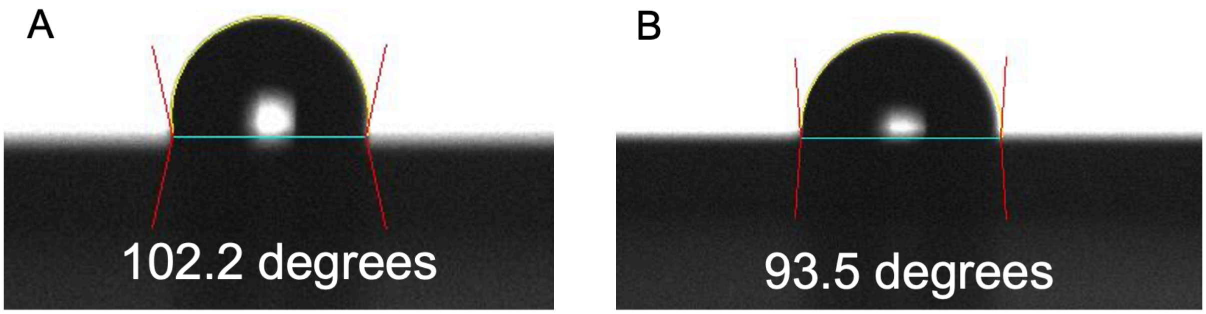

figure S3 Contact angle measurement. The water contact angle measurements before (A) and after (B) aptamer functionalization on the DPPTT/BA surface.

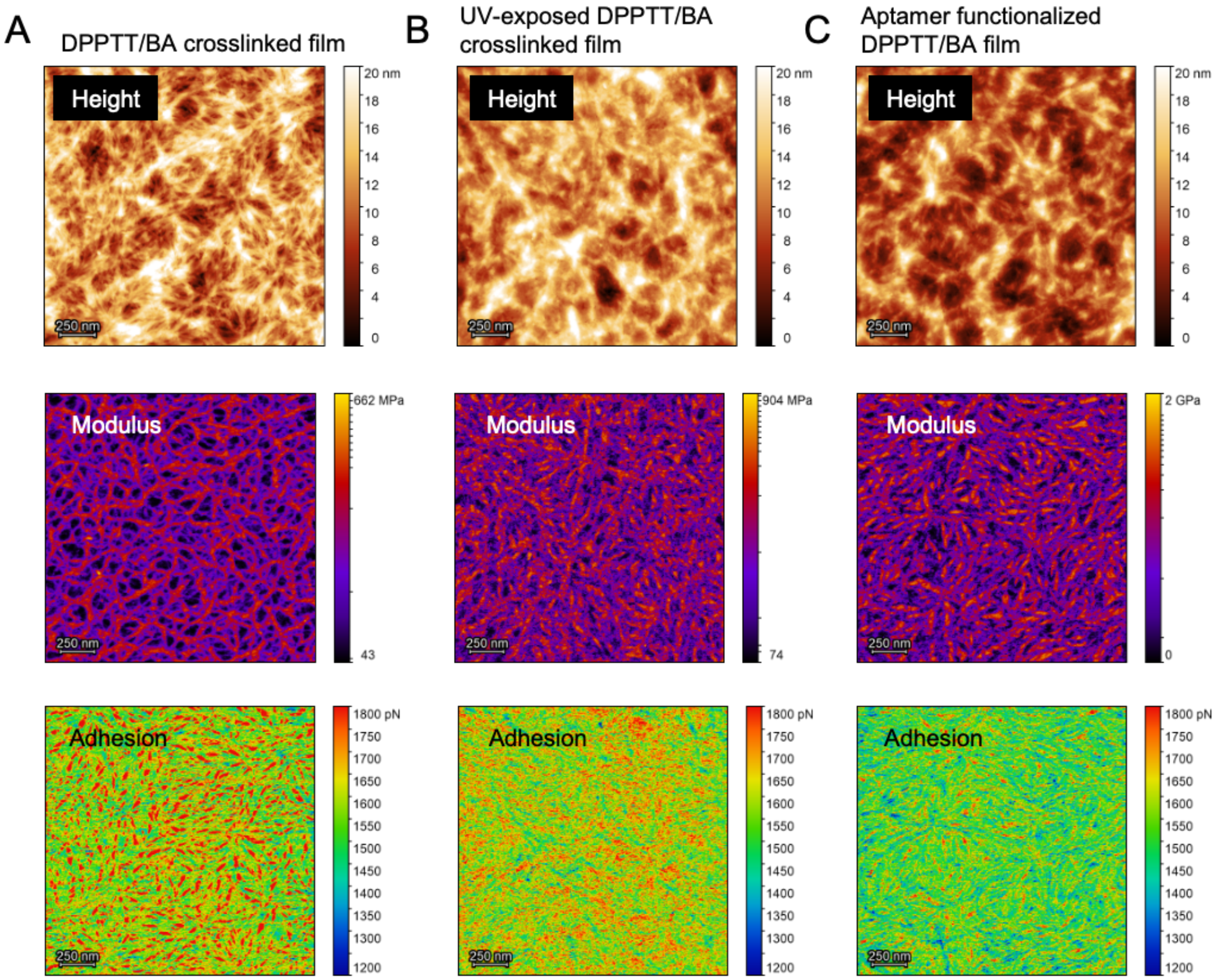

figure S4 Nanomechanical mapping of DPPTT/BA films. Nanomechanical mapping of height (top), DMT modulus (middle), and adhesion force (bottom) of DPPTT/BA crosslinked film (A), UV-exposed DPPTT/BA crosslinked film (B), and aptamer-functionalized DPPTT/BA crosslinked film (C) on OTS-modified SiO_2 substrates, respectively. Modulus and adhesion mapping images were reused, as in Figures 2D and 2F.

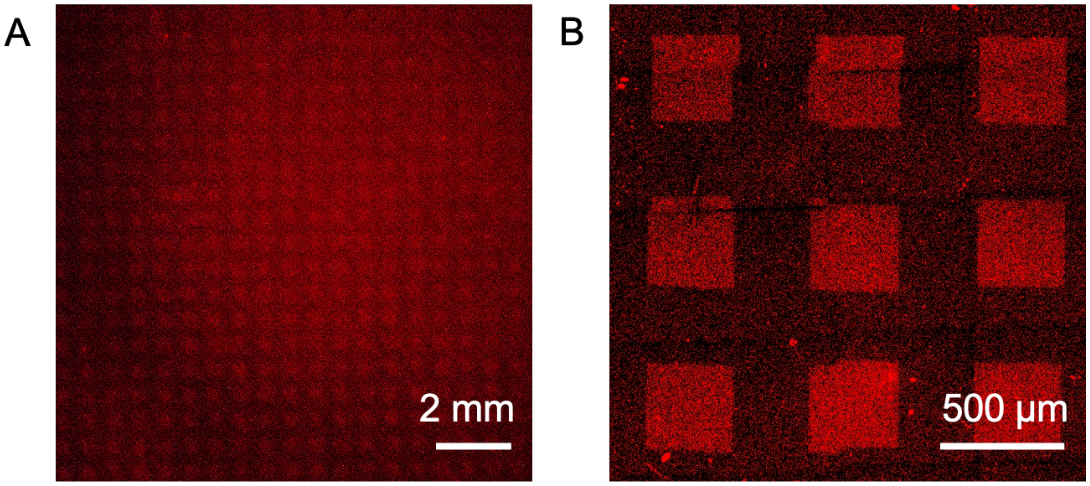

figure S5 Large-scale patterning of fluorophore-attached aptamers on DPPTT/BA surface.
(A, B) Fluorescence microscope images of Alexa-647-tagged aptamers at different scales.

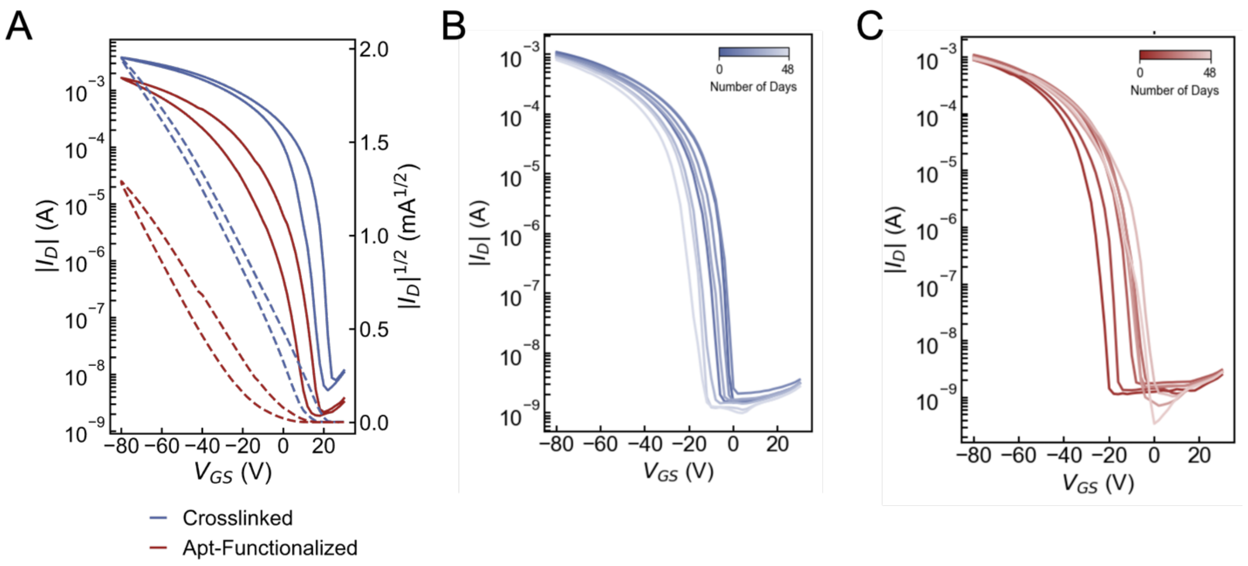

figure S6 Stability of solid-state OFETs in buffer. (A) Representative transfer curves of crosslinked DPPTT/BA and aptamer-functionalized BGTC transistors. Solid lines represent the I_D, and dotted lines represent the square root of the I_D. Transfer curves were collected at V_{DS} = –80 V. (B, C) Transfer curves of DPPTT/BA without and with aptamer functionalization in buffer for 48 days, respectively.

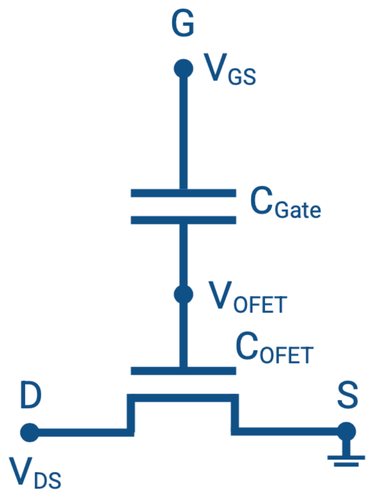

figure S7 Circuit diagram of EG-OFETs. In the diagram, C_{Gate} and C_{OFET} represent the capacitances at the gate and semiconductor interface, respectively. V_{GS} represents the applied voltage at the gate, and V_{OFET} represents the actual voltage dropped across the semiconductor interface.

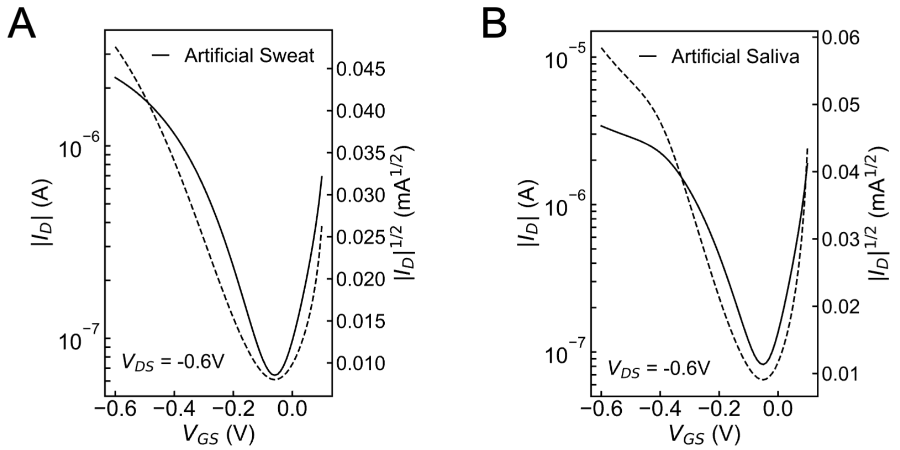

figure S8 Representative transfer curves of aptamer-functionalized EG-OFET. EG-OFETs were characterized with artificial sweat (A) and artificial saliva (B) as gate dielectric.

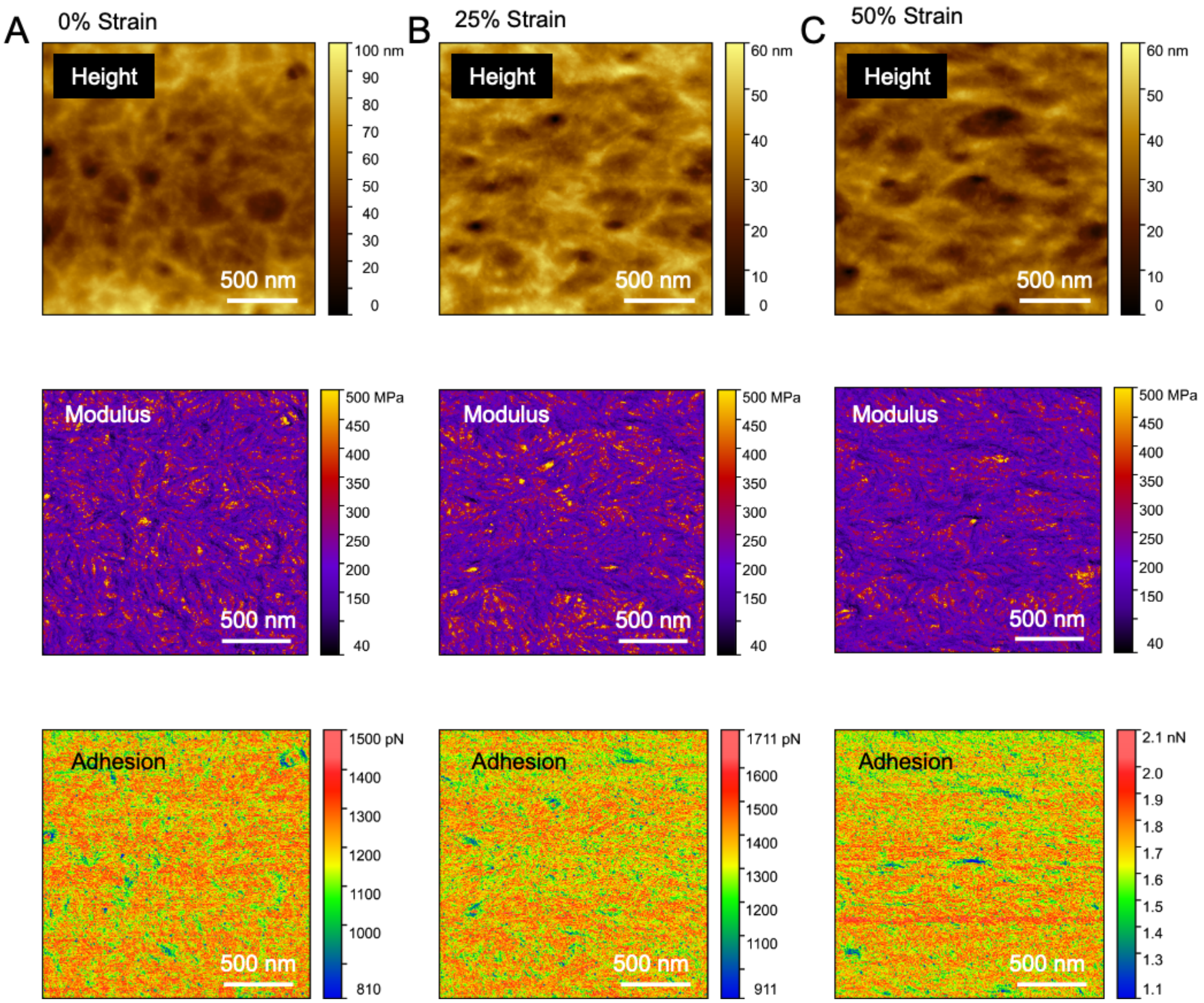

figure S9 Nanomechanical mapping of aptamer-functionalized DPPTT/BA crosslinked film under various mechanical strains. (A) 0% strain. (B) 25% strain. (C) 50% strain. Nanomechanical mapping of height, DMT modulus, and adhesion force of thermally crosslinked films on OTS-modified SiO_2 substrates. The modulus mapping image at 50% was reused, as in Figure 4E.

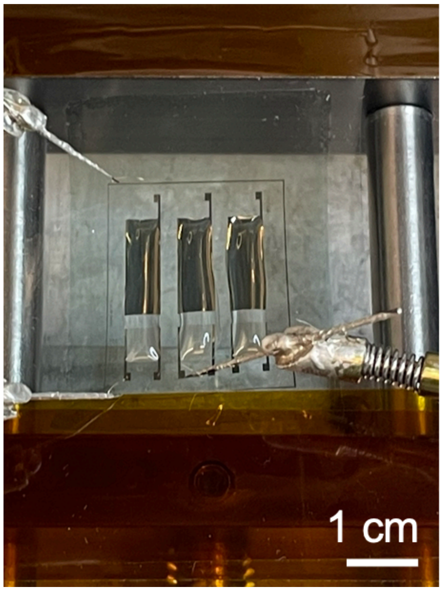

figure S10 Stretch measurement setup of EG-OFET in buffer. Photo showing the EG-OFET at a strain of 25% on a stretcher.

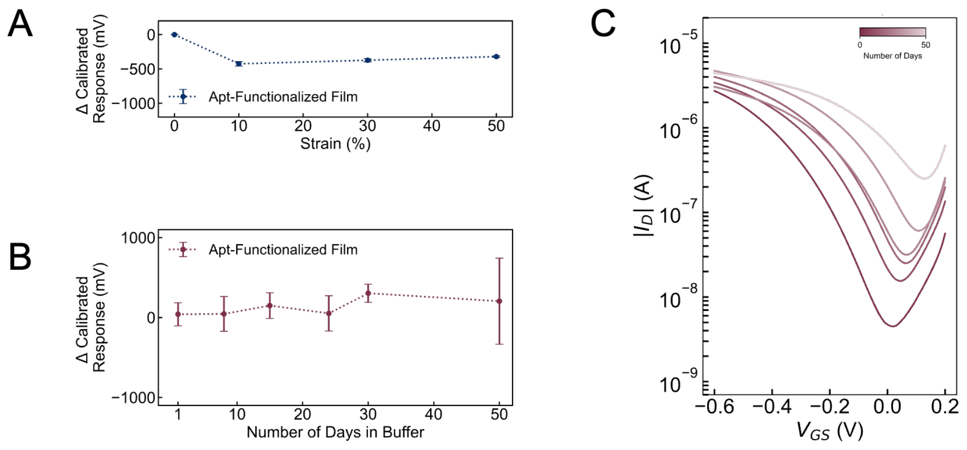

figure S11 Stability of aptamer-functionalized EG-OFETs during strain and when immersed in PBS buffer. (A) Changes in the calibrated response of soft EG-OFET when stretched up to 50%. Data are presented as mean values, and the error bars represent the standard deviation from 5 devices. (B) Changes in the calibrated response of aptamer-functionalized EG-OFETs immersed in buffer over 50 days. (C) Transfer curves of aptamer-functionalized DPPTT/BA in buffer for 50 days.

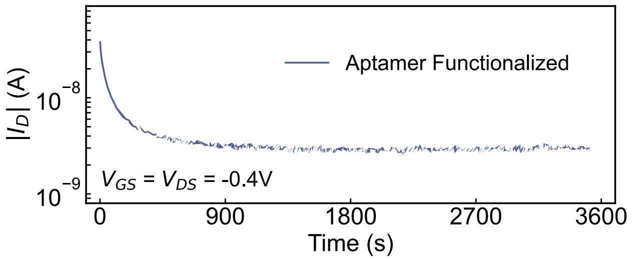

figure S12 Bias stress stability of aptamer-functionalized EG-OFETs. Transistors were under constant application of $V_{GS} = V_{DS} = –0.4V$ in air for 1 h.

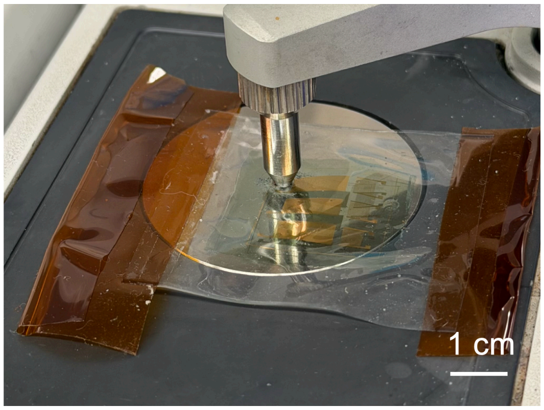

figure S13 Photo of measurement setup of surface-focused attenuated total reflective Fourier-transform infrared spectroscopy (ATR-FTIR).

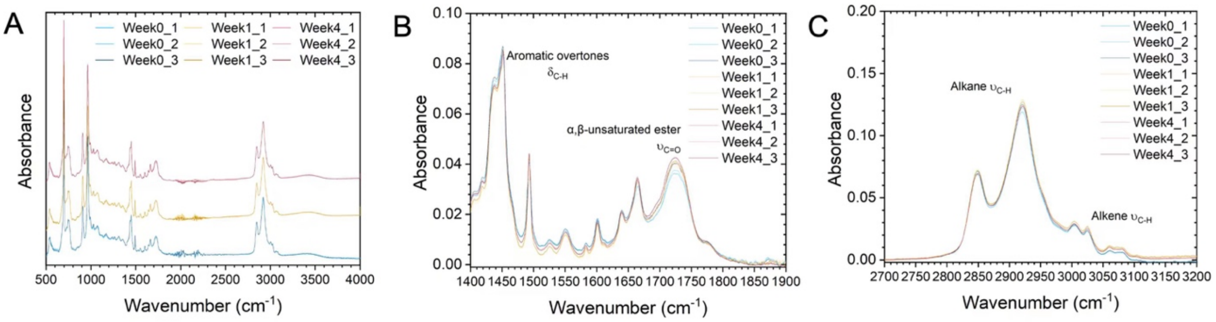

figure S14 ATR-FTIR spectra of aging tests of DPPTT/BA films stored in buffer solution for 4 weeks. (A) Full spectra and zoomed-in spectra of the C=O and C=C (B, C) regions.

Note: We performed surface-focused attenuated total reflective Fourier-transform infrared spectroscopy (ATR-FTIR) measurement of aptamer-functionalized DPPTT/BA on stretchable elastomers to study the chemical structure stability during the aging process for 4 weeks in buffer (fig. S13, S14). Identical chemical-bond characterization signals, in terms of bond-stretching frequencies and intensities, indicated no hydrolysis or oxidation under aging conditions (fig. S14). The signature peaks of C=O and C-H remain identical after aging in buffer solution.

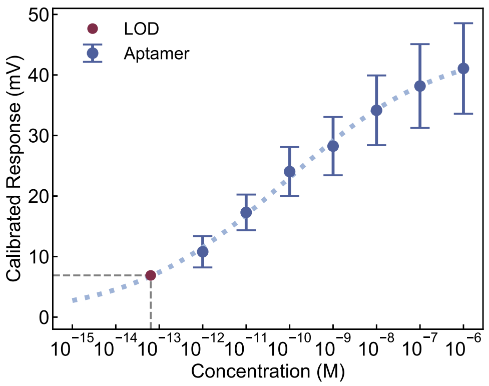

figure S15 Calibrated responses to cortisol for EG-OFETs functionalized with cortisol aptamer with LOD labeled.

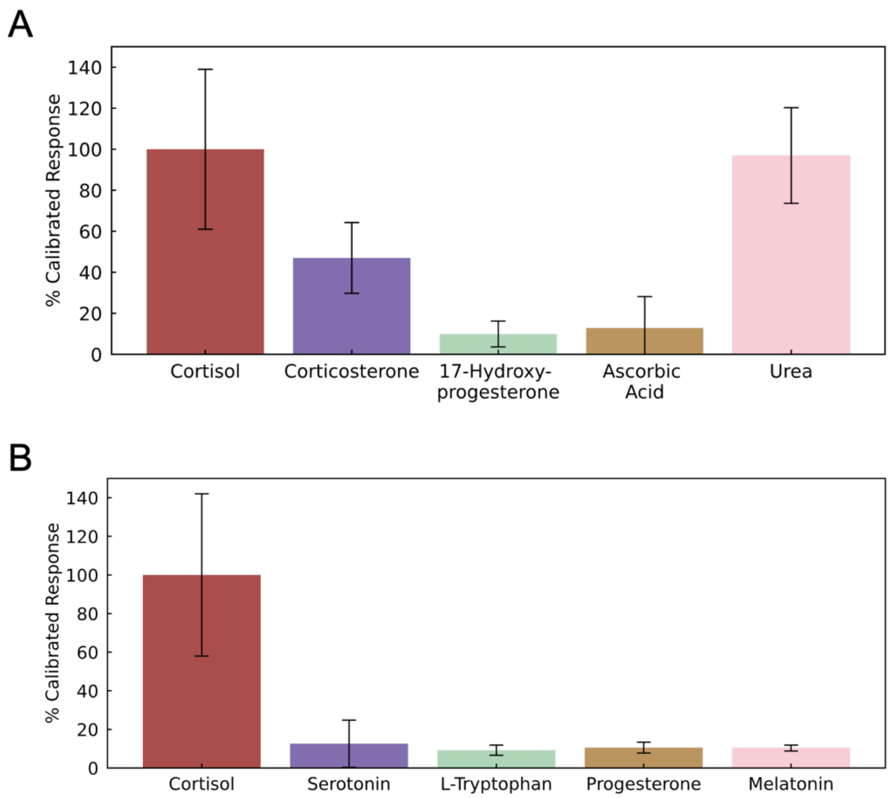

figure S16 Aptamer-functionalized EG-OFET responses to cortisol versus nontargets in buffer. (A) Cortisol, corticosterone, and 17-hydroxy-progesterone (10 nM), and ascorbic acid and Urea (20 mM) were measured in their physiological concentrations in sweat. (B) Cortisol, serotonin, L-tryptophan, progesterone, and melatonin (1nM) signals are directly compared using equal molarity to support Fig. 5E.

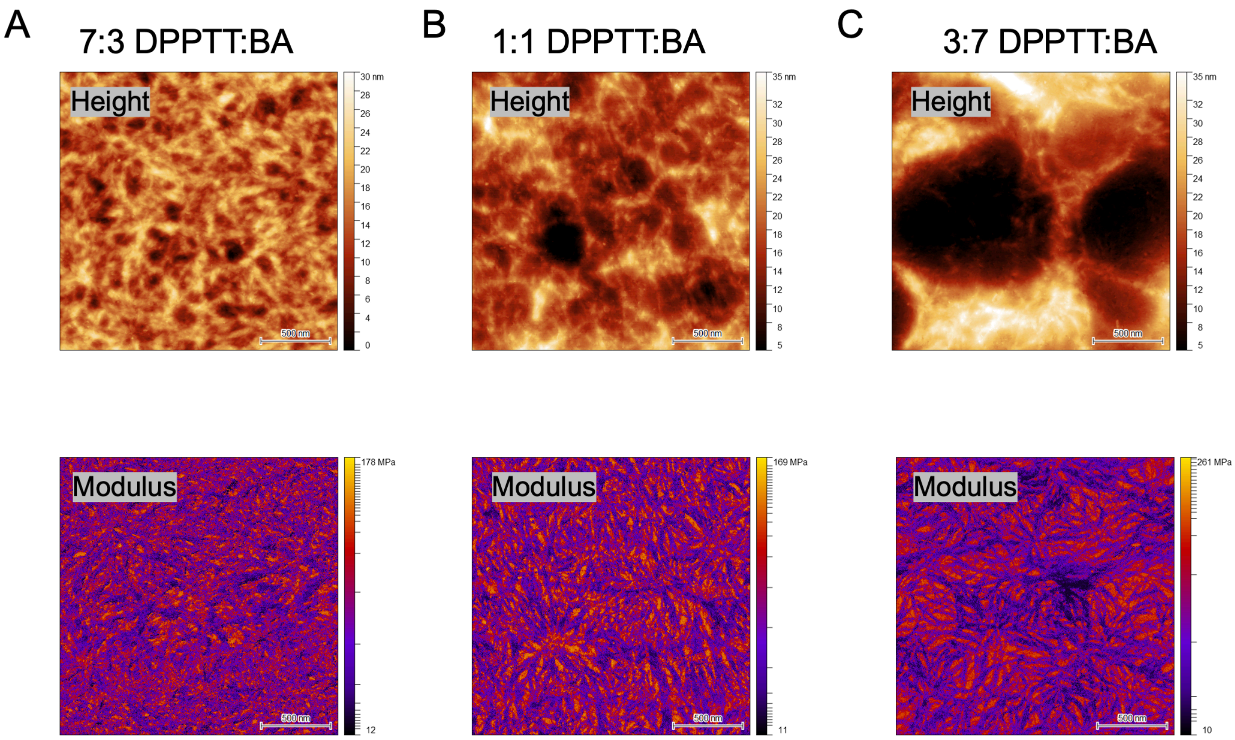

figure S17 Nanomechanical mapping of aptamer-functionalized DPPTT/BA crosslinked film with different DPPTT:BA ratio. (A) 7:3, (B) 1:1, and (C) 3:7.

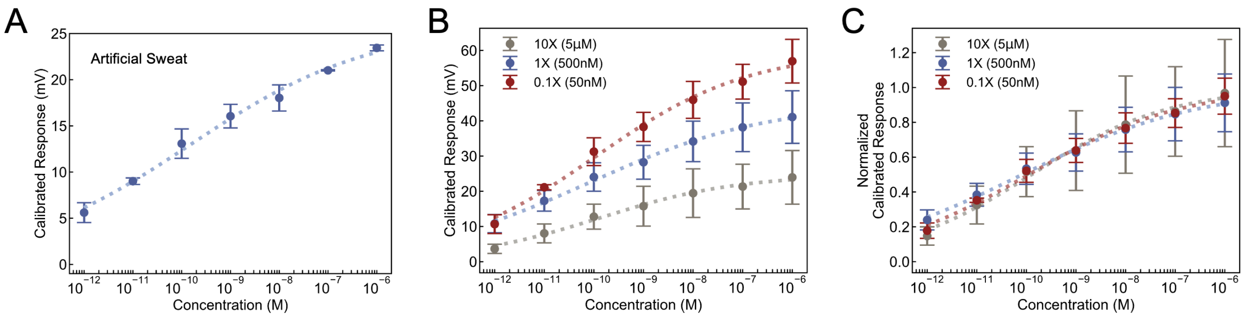

figure S18 Calibrated responses to cortisol for EG-OFETs functionalized with cortisol aptamer under various biological conditions and aptamer densities. (A) Artificial Sweat. (B,C) PBS with varying aptamer densities. Calibrated response (B) and normalized calibrated response (C) are reported.

REFERENCES

1. Y. Wang, H. Haick, S. Guo, C. Wang, S. Lee, T. Yokota, T. Someya, Skin bioelectronics towards long-term, continuous health monitoring. *Chem. Soc. Rev.* **51**, 3759–3793 (2022).

2. K. W. Cho, S.-H. Sunwoo, Y. J. Hong, J. H. Koo, J. H. Kim, S. Baik, T. Hyeon, D.-H. Kim, Soft bioelectronics based on nanomaterials. *Chem. Rev.* **122**, 5068–5143 (2022).

3. M. Lin, H. Hu, S. Zhou, S. Xu, Soft wearable devices for deep-tissue sensing. *Nat. Rev. Mater.* **7**, 850–869 (2022).

4. D. Khodagholy, T. Doublet, P. Quilichini, M. Gurfinkel, P. Leleux, A. Ghestem, E. Ismailova, T. Hervé, S. Sanaur, C. Bernard, G. G. Malliaras, In vivo recordings of brain activity using organic transistors. *Nat. Commun.* **4**, 1575 (2013).

5. C. Xu, S. A. Solomon, W. Gao, Artificial intelligence-powered electronic skin. *Nat. Mach. Intell.* **5**, 1344–1355 (2023).

6. J. Kim, S. Yoo, C. Liu, S. S. Kwak, J. R. Walter, S. Xu, J. A. Rogers, Skin-interfaced wireless biosensors for perinatal and paediatric health. *Nat. Rev. Bioeng.* **1**, 631–647 (2023).

7. T. Saha, R. Del Cano, K. Mahato, E. De la Paz, C. Chen, S. Ding, L. Yin, J. Wang, Wearable electrochemical glucose sensors in diabetes management: A comprehensive review. *Chem. Rev.* **123**, 7854–7889 (2023).

8. J. R. Sempionatto, J. A. Lasalde-Ramirez, K. Mahato, J. Wang, W. Gao, Wearable chemical sensors for biomarker discovery in the omics era. *Nat. Rev. Chem.* **6**, 899–915 (2022).

9. K. Sim, F. Ershad, Y. Zhang, P. Yang, H. Shim, Z. Rao, Y. Lu, A. Thukral, A. Elgalad, Y. Xi, B. Tian, D. A. Taylor, C. Yu, An epicardial bioelectronic patch made from soft rubbery materials and capable of spatiotemporal mapping of electrophysiological activity. *Nat. Electron.* **3**, 775–784 (2020).

10. N. Li, Y. Li, Z. Cheng, Y. Liu, Y. Dai, S. Kang, S. Li, N. Shan, S. Wai, A. Ziaja, Y. Wang, J. Strzalka, W. Liu, C. Zhang, X. Gu, J. A. Hubbell, B. Tian, S. Wang, Bioadhesive polymer semiconductors and transistors for intimate biointerfaces. *Science* **381**, 686–693 (2023).

11. C. Zhao, J. Park, S. E. Root, Z. Bao, Skin-inspired soft bioelectronic materials, devices and systems. *Nat. Rev. Bioeng.* **2**, 671–690 (2024).

12. S. Wang, J. Xu, W. Wang, G. N. Wang, R. Rastak, F. Molina-Lopez, J. W. Chung, S. Niu, V. R. Feig, J. Lopez, T. Lei, S. K. Kwon, Y. Kim, A. M. Foudeh, A. Ehrlich, A. Gasperini, Y. Yun, B. Murmann, J. B. Tok, Z. Bao, Skin electronics from scalable fabrication of an intrinsically stretchable transistor array. *Nature* **555**, 83–88 (2018).

13. W. Wang, Y. Jiang, D. Zhong, Z. Zhang, S. Choudhury, J.-C. Lai, H. Gong, S. Niu, X. Yan, Y. Zheng, C.-C. Shih, R. Ning, Q. Lin, D. Li, Y.-H. Kim, J. Kim, Y.-X. Wang, C. Zhao, C. Xu, X. Ji, Y. Nishio, H. Lyu, J. B. H. Tok, Z. Bao, Neuromorphic sensorimotor loop embodied by monolithically integrated, low-voltage, soft e-skin. *Science* **380**, 735–742 (2023).

14. S. Yuvaraja, A. Nawaz, Q. Liu, D. Dubal, S. G. Surya, K. N. Salama, P. Sonar, Organic field-effect transistor-based flexible sensors. *Chem. Soc. Rev.* **49**, 3423–3460 (2020).

15. Y. Dai, H. Hu, M. Wang, J. Xu, S. Wang, Stretchable transistors and functional circuits for human-integrated electronics. *Nat. Electron.* **4**, 17–29 (2021).

16. M. Sugiyama, T. Uemura, M. Kondo, M. Akiyama, N. Namba, S. Yoshimoto, Y. Noda, T. Araki, T. Sekitani, An ultraflexible organic differential amplifier for recording electrocardiograms. *Nat. Electron.* **2**, 351–360 (2019).

17. M. Kondo, M. Melzer, D. Karnaushenko, T. Uemura, S. Yoshimoto, M. Akiyama, Y. Noda, T. Araki, O. G. Schmidt, T. Sekitani, Imperceptible magnetic sensor matrix system integrated with organic driver and amplifier circuits. *Sci. Adv.* **6**, eaay6094 (2020).

18. Y. Dai, S. Wai, P. Li, N. Shan, Z. Cao, Y. Li, Y. Wang, Y. Liu, W. Liu, K. Tang, Y. Liu, M. Hua, S. Li, N. Li, S. Chatterji, H. C. Fry, S. Lee, C. Zhang, M. Weires, S. Sutyak, J. Shi, C. Zhu,

J. Xu, X. Gu, B. Tian, S. Wang, Soft hydrogel semiconductors with augmented biointeractive functions. *Science* **386**, 431–439 (2024).

19. N. Li, Y. Dai, Y. Li, S. Dai, J. Strzalka, Q. Su, N. De Oliveira, Q. Zhang, P. B. J. St. Onge, S. Rondeau-Gagné, Y. Wang, X. Gu, J. Xu, S. Wang, A universal and facile approach for building multifunctional conjugated polymers for human-integrated electronics. *Matter* **4**, 3015–3029 (2021).

20. W. Wu, S. Yu, D. S. Forbes, H. Jiang, M. Ahmed, J. Mei, Efficient and modular biofunctionalization of thiophene-based conjugated polymers through embedded latent disulfide. *J. Am. Chem. Soc.* **146**, 578–585 (2024).

21. Z. Jiang, D. Ye, L. Xiang, Z. He, X. Dai, J. Yang, Q. Xiong, Y. Ma, D. Zhi, Y. Zou, Q. Peng, S. Wang, J. Li, F. Zhang, C. A. Di, A drug-mediated organic electrochemical transistor for robustly reusable biosensors. *Nat. Mater.* **23**, 1547–1555 (2024).

22. N. Li, S. Kang, Z. Liu, S. Wai, Z. Cheng, Y. Dai, A. Solanki, S. Li, Y. Li, J. Strzalka, M. J. V. White, Y. H. Kim, B. Tian, J. A. Hubbell, S. Wang, Immune-compatible designs of semiconducting polymers for bioelectronics with suppressed foreign-body response. *Nat. Mater.* **25**, 124–132 (2026).

23. F. Torricelli, D. Z. Adrahtas, Z. Bao, M. Berggren, F. Biscarini, A. Bonfiglio, C. A. Bortolotti, C. D. Frisbie, E. Macchia, G. G. Malliaras, I. McCulloch, M. Moser, T. Q. Nguyen, R. M. Owens, A. Salleo, A. Spanu, L. Torsi, Electrolyte-gated transistors for enhanced performance bioelectronics. *Nat. Rev. Methods Primers* **1**, 66 (2021).

24. R. A. Picca, K. Manoli, E. Macchia, L. Sarcina, C. Di Franco, N. Cioffi, D. Blasi, R. Österbacka, F. Torricelli, G. Scamarcio, L. Torsi, Ultimately sensitive organic bioelectronic transistor sensors by materials and device structure design. *Adv. Funct. Mater.* **30**, 1904513 (2020).

25. S. P. White, K. D. Dorfman, C. D. Frisbie, Operating and sensing mechanism of electrolyte-gated transistors with floating gates: Building a platform for amplified biodetection. *J. Phys. Chem. C* **120**, 108–117 (2016).

26. P. Seshadri, K. Manoli, N. Schneiderhan-Marra, U. Anthes, P. Wierzchowiec, K. Bonrad, C. Di Franco, L. Torsi, Low-picomolar, label-free procalcitonin analytical detection with an electrolyte-gated organic field-effect transistor based electronic immunosensor. *Biosens. Bioelectron.* **104**, 113–119 (2018).

27. A. Haque, K. M. Alenezi, M. S. Khan, W. Y. Wong, P. R. Raithby, Non-covalent interactions (NCIs) in pi-conjugated functional materials: Advances and perspectives. *Chem. Soc. Rev.* **52**, 454–472 (2023).

28. Y. Jiang, D. Ohayon, B. R. P. Yip, G. Quek, Z. Chen, G. C. Bazan, Conjugated polyelectrolyte-aptamer hybrid for organic-electrochemical-transistor-based sensing. *Cell Rep. Phys. Sci.* **6**, 102965 (2025).

29. C. Y. Xue, K. L. Yang, Chemical modifications of inert organic monolayers with oxygen plasma for biosensor applications. *Langmuir* **23**, 5831–5835 (2007).

30. F. Werkmeister, B. Nickel, Towards flexible organic thin film transistors (OTFTs) for biosensing. *J. Mater. Chem. B* **1**, 3830–3835 (2013).

31. J. Xu, S. Wang, G. N. Wang, C. Zhu, S. Luo, L. Jin, X. Gu, S. Chen, V. R. Feig, J. W. To, S. Rondeau-Gagne, J. Park, B. C. Schroeder, C. Lu, J. Y. Oh, Y. Wang, Y. H. Kim, H. Yan, R. Sinclair, D. Zhou, G. Xue, B. Murmann, C. Linder, W. Cai, J. B. Tok, J. W. Chung, Z. Bao, Highly stretchable polymer semiconductor films through the nanoconfinement effect. *Science* **355**, 59–64 (2017).

32. Y. Zheng, Z. Yu, S. Zhang, X. Kong, W. Michaels, W. Wang, G. Chen, D. Liu, J.-C. Lai, N. Prine, W. Zhang, S. Nikzad, C. B. Cooper, D. Zhong, J. Mun, Z. Zhang, J. Kang, J. B. H. Tok, I. McCulloch, J. Qin, X. Gu, Z. Bao, A molecular design approach towards elastic and multifunctional polymer electronics. *Nat. Commun.* **12**, 5701 (2021).

33. Y. Liu, J. Canoura, O. Alkhamis, Y. Xiao, Immobilization strategies for enhancing sensitivity of electrochemical aptamer-based sensors. *ACS Appl. Mater. Interfaces* **13**, 9491–9499 (2021).

34. Y. Liang, R. A. van der Valk, R. T. Dame, W. H. Roos, G. J. L. Wuite, Probing the mechanical stability of bridged DNA-h-ns protein complexes by single-molecule AFM pulling. *Sci. Rep.* **7**, 15275 (2017).

35. E. Delamarche, I. Pereiro, A. Kashyap, G. V. Kaigala, Biopatterning: The art of patterning biomolecules on surfaces. *Langmuir* **37**, 9637–9651 (2021).

36. I. Batalov, K. R. Stevens, C. A. DeForest, Photopatterned biomolecule immobilization to guide three-dimensional cell fate in natural protein-based hydrogels. *Proc. Natl. Acad. Sci. U.S.A.* **118**, e2014194118 (2021).

37. P. Bandaru, D. Chu, W. Sun, S. Lasli, C. Zhao, S. Hou, S. Zhang, J. Ni, G. Cefaloni, S. Ahadian, M. R. Dokmeci, S. Sengupta, J. Lee, A. Khademhosseini, A microfabricated sandwiching assay for nanoliter and high-throughput biomarker screening. *Small* **15**, e1900300 (2019).

38. S. A. Lange, V. Benes, D. P. Kern, J. K. Horber, A. Bernard, Microcontact printing of DNA molecules. *Anal. Chem.* **76**, 1641–1647 (2004).

39. M. J. Shuster, A. Vaish, H. H. Cao, A. I. Guttentag, J. E. McManigle, A. L. Gibb, M. Martinez-Rivera, R. M. Nezarati, J. M. Hinds, W.-S. Liao, P. S. Weiss, A. M. Andrews, Patterning small-molecule biocapture surfaces: Microcontact insertion printing vs. photolithography. *Chem. Comm.* **47**, 10641–10643 (2011).

40. J. Kim, Y. S. Rim, H. Chen, H. H. Cao, N. Nakatsuka, H. L. Hinton, C. Zhao, A. M. Andrews, Y. Yang, P. S. Weiss, Fabrication of high-performance ultrathin In2O3 film field-effect transistors and biosensors using chemical lift-off lithography. *ACS Nano* **9**, 4572–4582 (2015).

41. O. Knopfmacher, M. L. Hammock, A. L. Appleton, G. Schwartz, J. Mei, T. Lei, J. Pei, Z. Bao, Highly stable organic polymer field-effect transistor sensor for selective detection in the marine environment. *Nat. Commun.* **5**, 2954 (2014).

42. Y. Zheng, L. Michalek, Q. Liu, Y. Wu, H. Kim, P. Sayavong, W. Yu, D. Zhong, C. Zhao, Z. Yu, J. A. Chiong, H. Gong, X. Ji, D. Liu, S. Zhang, N. Prine, Z. Zhang, W. Wang, J. B. Tok, X. Gu, Y. Cui, J. Kang, Z. Bao, Environmentally stable and stretchable polymer electronics enabled by surface-tethered nanostructured molecular-level protection. *Nat. Nanotechnol.* **18**, 1175–1184 (2023).

43. L. Torsi, M. Magliulo, K. Manoli, G. Palazzo, Organic field-effect transistor sensors: A tutorial review. *Chem. Soc. Rev.* **42**, 8612–8628 (2013).

44. E. Macchia, K. Manoli, B. Holzer, C. Di Franco, M. Ghittorelli, F. Torricelli, D. Alberga, G. F. Mangiatordi, G. Palazzo, G. Scamarcio, L. Torsi, Single-molecule detection with a millimetre-sized transistor. *Nat. Commun.* **9**, 3223 (2018).

45. N. Nakatsuka, K.-A. Yang, J. M. Abendroth, K. M. Cheung, X. Xu, H. Yang, C. Zhao, B. Zhu, Y. S. Rim, Y. Yang, P. S. Weiss, M. N. Stojanović, A. M. Andrews, Aptamer–field-effect transistors overcome debye length limitations for small-molecule sensing. *Science* **362**, 319–324 (2018).

46. N. Matsuhisa, Y. Jiang, Z. Liu, G. Chen, C. Wan, Y. Kim, J. Kang, H. Tran, H. C. Wu, I. You, Z. Bao, X. Chen, High-transconductance stretchable transistors achieved by controlled gold microcrack morphology. *Adv. Electron. Mater.* **5**, 1900347 (2019).

47. Z. Jiang, N. Chen, Z. Yi, J. Zhong, F. Zhang, S. Ji, R. Liao, Y. Wang, H. Li, Z. Liu, Y. Wang, T. Yokota, X. Liu, K. Fukuda, X. Chen, T. Someya, A 1.3-micrometre-thick elastic conductor for seamless on-skin and implantable sensors. *Nat. Electron.* **5**, 784–793 (2022).

48. D. Simatos, M. Nikolka, J. Charmet, L. J. Spalek, Z. Toprakcioglu, I. E. Jacobs, I. B. Dimov, G. Schweicher, M. J. Lee, C. M. Fernández-Posada, D. J. Howe, T. A. Hakala, L. W. Y. Roode, V. Pecunia, T. P. Sharp, W. Zhang, M. Alsufyani, I. McCulloch, T. P. J. Knowles, H. Sirringhaus, Electrolyte-gated organic field-effect transistors with high operational stability and lifetime in practical electrolytes. *SmartMat* **5**, e1291 (2024).

49. C. Zhao, K. M. Cheung, I. W. Huang, H. Yang, N. Nakatsuka, W. Liu, Y. Cao, T. Man, P. S. Weiss, H. G. Monbouquette, A. M. Andrews, Implantable aptamer–field-effect transistor neuroprobes for in vivo neurotransmitter monitoring. *Sci. Adv.* **7**, eabj7422 (2021).

50. B. Wang, C. Zhao, Z. Wang, K.-A. Yang, X. Cheng, W. Liu, W. Yu, S. Lin, Y. Zhao, K. M. Cheung, H. Lin, H. Hojaiji, P. S. Weiss, M. N. Stojanović, A. J. Tomiyama, A. M. Andrews, S. Emaminejad, Wearable aptamer-field-effect transistor sensing system for noninvasive cortisol monitoring. *Sci. Adv.* **8**, eabk0967 (2022).

51. K. M. Cheung, K. A. Yang, N. Nakatsuka, C. Zhao, M. Ye, M. E. Jung, H. Yang, P. S. Weiss, M. N. Stojanovic, A. M. Andrews, Phenylalanine monitoring via aptamer-field-effect transistor sensors. *ACS Sens.* **4**, 3308–3317 (2019).

52. H. J. Jang, T. Lee, J. Song, L. Russell, H. Li, J. Dailey, P. C. Searson, H. E. Katz, Electronic cortisol detection using an antibody-embedded polymer coupled to a field-effect transistor. *ACS Appl. Mater. Interfaces* **10**, 16233–16237 (2018).

53. O. Parlak, S. T. Keene, A. Marais, V. F. Curto, A. Salleo, Molecularly selective nanoporous membrane-based wearable organic electrochemical device for noninvasive cortisol sensing. *Sci. Adv.* **4**, eaar2904 (2018).

54. C. Zhao, J. Park, D. Maulà, Y. Yuan, D. Zhong, W. Wang, Q. Liu, C. Xu, Y. Zheng, R. K. Mow, Y. Jiang, C. Xu, H. Lyu, L. Michalek, A. Berman, Y. Jiang, S. Wei, C. Zhu, C. Wu, A. Abramson, E. Kim, X. Ji, Z. Yu, J. Shi, M. Khatib, B. Shi, Z. Bao, Skin-like drift-free biosensors with stretchable diode-connected organic field-effect transistors. *Nat. Electron.* **8**, 981–993 (2025).

55. E. Kim, A. L. Ramos Figueroa, M. Schrock, E. Zhang, C. J. Newcomb, Z. Bao, L. Michalek, A guide for nanomechanical characterization of soft matter via AFM: From mode selection to data reporting. *STAR Protoc.* **6**, 103809 (2025).

56. Y. Q. Zheng, Z. Bao, Molecularly designed and nanoconfined polymer electronic materials for skin-like electronics. *ACS Cent. Sci.* **10**, 2188–2199 (2024).

57. N. Maganzini, I. Thompson, B. Wilson, H. T. Soh, Pre-equilibrium biosensors as an approach towards rapid and continuous molecular measurements. *Nat. Commun.* **13**, 7072 (2022).
